# Macrophages Derived From Human Induced Pluripotent Stem Cells: The Diversity of Protocols, Future Prospects, and Outstanding Questions

**DOI:** 10.3389/fcell.2021.640703

**Published:** 2021-06-02

**Authors:** Irina Lyadova, Tatiana Gerasimova, Tatiana Nenasheva

**Affiliations:** Laboratory of Cellular and Molecular Basis of Histogenesis, Koltzov Institute of Developmental Biology of the Russian Academy of Sciences, Moscow, Russia

**Keywords:** macrophages, iPSC-derived macrophages, macrophage differentiation, *in vitro* protocols, culture condition optimization, interleukin-3, M-CSF

## Abstract

Macrophages (Mφ) derived from induced pluripotent stem cells (iMphs) represent a novel and promising model for studying human Mφ function and differentiation and developing new therapeutic strategies based on or oriented at Mφs. iMphs have several advantages over the traditionally used human Mφ models, such as immortalized cell lines and monocyte-derived Mφs. The advantages include the possibility of obtaining genetically identical and editable cells in a potentially scalable way. Various applications of iMphs are being developed, and their number is rapidly growing. However, the protocols of iMph differentiation that are currently used vary substantially, which may lead to differences in iMph differentiation trajectories and properties. Standardization of the protocols and identification of minimum required conditions that would allow obtaining iMphs in a large-scale, inexpensive, and clinically suitable mode are needed for future iMph applications. As a first step in this direction, the current review discusses the fundamental basis for the generation of human iMphs, performs a detailed analysis of the generalities and the differences between iMph differentiation protocols currently employed, and discusses the prospects of iMph applications.

## Introduction

Macrophages (Mφs) are innate immune cells involved in fundamental biological processes, including inflammation development and homeostasis support. They mediate host protection by engulfing and eliminating pathogens, by secreting a wide range of proinflammatory mediators that attract and activate immune cells at the site of infection, and by processing and presenting antigens to T lymphocytes, which propagates an adaptive immune response in the tissues (Wynn et al., 2013; Duque and Descoteaux, 2014; Weiss and Schaible, 2015). Mφs are also able to limit inflammation and mediate tissue repair and wound healing, largely by secreting anti-inflammatory and tissue remodeling factors and by phagocytizing apoptotic and necrotic cells (Mantovani et al., 2013; Wynn and Vannella, 2016; Hamidzadeh et al., 2017; Galloway et al., 2019; Watanabe et al., 2019). The foundation for the manifold and often opposite activities is formed by Mφ capacity to sense the microenvironment and fine-tune their transcriptomic and functional programs according to homeostatic requirements. Dysregulation of these processes underlies many diseases. In particular, an exacerbated inflammatory response and/or impaired phagocytic/clearance activities of Mφs have been implicated in the pathogenesis of autoimmune, chronic inflammatory, cardiovascular, metabolic, neurodegenerative, infectious, and several hereditary diseases (Lyadova, 2012; Byrne et al., 2015; Ma et al., 2018; Parisi et al., 2018; Ardura et al., 2019; Galloway et al., 2019; Trapnell et al., 2019; Merad and Martin, 2020). In turn, insufficient inflammatory potential and/or excessive secretion of anti-inflammatory and tissue remodeling mediators induce fibrosis and promote cancer initiation, invasion, and metastasis (Wynn and Vannella, 2016; J.W. Cassetta and Pollard, 2018; Guerrini and Gennaro, 2019). Thus, Mφs represent an attractive therapeutic target. However, to develop Mφ-oriented therapeutic strategies, adequate Mφ models are needed that allow to unravel the mechanisms regulating Mφ activity, to model pathological conditions, and to perform drug testing.

Macrophages reside and execute their functions in peripheral tissues. Consequently, it is of primary interest to model tissue resident Mφs (TRMs). van Furth and Cohn (1968) demonstrated that blood monocytes originate from bone marrow (BM) progenitor cells and, in response to sterile inflammation, enter the peritoneal cavity and give rise to peritoneal Mφs. The life history of mononuclear phagocyte cells was formulated to be as follows: BM promonocytes → peripheral blood monocytes → Mφs in the tissues; the concept of a single mononuclear phagocyte system that unites BM progenitors, blood monocytes, and Mφs was suggested (van Furth et al., 1972; Gordon and Taylor, 2005; Hume, 2006). Later studies performed in mice demonstrated that some TRMs arise during the early embryonic period independently of BM hematopoiesis; the cells seed the tissues prior to birth, self-renew, and maintain locally (Ginhoux et al., 2010; Schulz et al., 2012; Guilliams et al., 2013; Hashimoto et al., 2013; Yona et al., 2013; Hoeffel et al., 2015). Throughout the lifetime, in some tissues and/or in inflammatory conditions, TRMs of embryonic origin are replenished by monocyte-derived Mφs (MDMs) (Bain et al., 2014; Epelman et al., 2014; Jenkins and Hume, 2014; Molawi et al., 2014; Coillard and Segura, 2019; Hume et al., 2019). Nevertheless, in most tissues, MDMs form only a minor part of TRMs (Ginhoux and Guilliams, 2016; Mildner et al., 2016; De Schepper et al., 2018), and this should be considered when modeling Mφs.

Until recently, there were a limited number of approaches available for the analysis of human Mφs, and none of them modeled TRMs. Lately, methods of Mφ differentiation from pluripotent stem cells (PSCs), first from embryonic stem cells (ESCs) and later from induced pluripotent stem cells (iPSCs), have been elaborated and began to be widely used. The methods used in different laboratories share the same general principle of a stepwise differentiation of ESCs/iPSCs into Mφs (hereafter referred to as iMphs) through the formation of mesoderm, hemogenic endothelium (HE), hematopoietic progenitors, and monocytic cells. However, the details of the protocols vary substantially, which may affect the efficiency of iMph generation, cell differentiation trajectories, and iMph biological properties. Here, we consider the fundamental basis for iMph generation, review the generalities of and the differences between distinct iMph differentiation protocols, and discuss the prospects of iMph applications, focusing primarily on the generation of Mφs from human iPSCs.

## Models Used for Human Mφ Studies

### Direct Isolation of TRMs

Direct isolation of TRMs from the tissues would be the most relevant model for Mφ analysis; however, it is limited because of poor availability of human tissues. Animal TRMs do not help to overcome the limitation, as there are significant interspecies differences in Mφ transcriptomic, metabolic, and functional programs (Weinberg, 1998; Albina and Reichner, 2003; Schneemann and Schoeden, 2007; Vijayan et al., 2019). Moreover, Mφs cannot be obtained in sufficient quantities from most tissues, even in animals. The use of activation and/or mobilizing stimuli may help to increase cell yield (the classical example is the intraperitoneal injection of pepton to mobilize peritoneal mouse Mφs Zhang X. et al., 2008), but this method, as well as tissue disaggregation and separation (Summers et al., 2020), affects cell activity, making the analysis of steady-state “naive” TRMs impossible.

### Immortalized Cell Lines

Immortalized cell lines, such as THP-1 or U937, constitute the easiest to handle human Mφ model. The cells originate from hemato-oncological patients and contain highly proliferative suspensive CD14^+^ “monocyte-like” cells that can be differentiated into “Mφ-like” cells by culturing them in the presence of stimulating (phorbol myristate acetate) or differentiating [e.g., Mφ colony-stimulating factor (M-CSF)] stimuli (Rodell et al., 2019). The approach has significant technical advantages; specifically, the cells are robust and highly proliferative and can be genetically manipulated, and their maintenance and expansion are technically easy and cheap. However, the biological relevance of these cell lines is limited, as the cells have a unique genetic background, derive from malignant cells, and cannot adequately model nature monocytes/Mφs and their genetic diversity (Bosshart and Heinzelmann, 2016).

### MDMs

The generation of MDMs is the most widely used approach for generating human Mφs. In this approach, CD14^+^ monocytes isolated from peripheral blood mononuclear cells are treated with cytokines/growth factors (most often with M-CSF) to generate Mφs (Brugger et al., 1991; Plesner, 2003). In experimental settings, a similar model uses BM cells as a source for generating Mφs (Trouplin et al., 2013). Considering the concept of a single mononuclear phagocyte system, both models have long been regarded as the most relevant ones. The important advantages of the MDM model are the easy accessibility of human peripheral blood samples and the possibility of obtaining up to several millions of MDMs from one donor. However, MDMs do not proliferate and cannot be maintained in culture for a prolonged period; they are scarce and difficult to access from patients with rare diseases and to be genetically modified [although the first success was recently achieved by Klichinsky et al. (2020)]. Another limitation, which is a fundamental one, is that MDMs cannot fully model TRMs (discussed above and in the references Ginhoux et al., 2010; Schulz et al., 2012; Guilliams et al., 2013; Hashimoto et al., 2013; Yona et al., 2013; Hoeffel et al., 2015).

### Mφs Generated *in vitro* From Pluripotent Stem Cells

To overcome the limitations of existing human Mφ models, methods of generating Mφs from PSCs have recently been developed. In this approach, ESCs or iPSCs are cultured in conditions that drive cell differentiation through the pathway that recapitulates embryonic hematopoiesis; the resulting cells (iMphs) were suggested to be a better model of TRMs compared to MDMs (Buchrieser et al., 2017; Takata et al., 2017; Lee et al., 2018; Tasnim et al., 2019). Other advantages of the method include an easy availability of PSCs and scalability, standardizability, and the possibility of generating genetically manipulated cells (Yeung et al., 2017; Zhang et al., 2017; Klatt et al., 2019), which altogether significantly expands possible applications of the model.

## The Generation of Mφs During Embryonic Hematopoiesis

**FIGURE 1:**
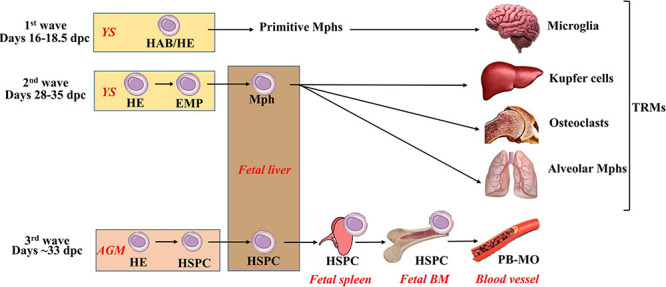
Three waves of macrophage generation during embryonic hematopoiesis. Embryonic hematopoiesis occurs in at least three waves. The first wave (primitive hematopoiesis) takes place extraembryonically in the yolk sac. At this wave, primitive macrophages, erythrocytes, and megakaryocytes are generated. Their exact cellular source is not fully clear: it is suggested that this is HAB (i.e., a common precursor of hematopoietic and endothelial cells), but the emergence of primitive hematopoietic cells directly from mesoderm or HE (i.e., endothelial cells having a potential to generate blood cells) is also considered. Primitive macrophages migrate to the central nervous system to form primitive microglia, and they also give rise to Langerhans cells. During the second wave (early or the first definitive), HE is formed and generates EMPs that give rise to definitive erythrocytes, megakaryocytes, and myeloid cells. EMP-derived macrophages mature in the fetal liver, seed the tissues (other than brain), and form self-renewing TRM pools. The third (definitive) wave takes place primarily in the AGM (other involved sites include placenta, vitelline, and umbilical arteries). At this stage, HE gives rise to long-term repopulating HSPCs. HSPCs migrate to the fetal liver; self-renew; expand; home to the spleen, thymus, and fetal BM and initiate adult-type hematopoiesis. AGM, aorta-gonad mesonephros; HAB, hemangioblast; HE, hemogenic endothelium; HSPCs, hematopoietic stem and progenitor cells; EMP, erythromyeloid progenitors; PB-MO, peripheral blood monocytes.

To understand the principles of iMph differentiation, it is important to briefly review the pathways of Mφ differentiation during embryogenesis (Figure 1). These were best studied in animal models (reviewed in detail in Dzierzak and Speck, 2008; Medvinsky et al., 2011; McGrath et al., 2015; Yumine et al., 2017; Dzierzak and Bigas, 2018; Hadland and Yoshimoto, 2018; Laurenti and Göttgens, 2018; Yamane, 2018).

Embryonic hematopoiesis is divided into primitive (fetal) and definitive (adult-like), and it occurs in at least three waves. In all waves, hematopoietic differentiation starts with the formation of cells expressing endothelium markers that give rise to different types of hematopoietic cells.

The first wave, also called primitive hematopoiesis, takes place extraembryonically in the yolk sac [YS; E7.0–9.0 in mice; 16–18.5 days postconception (dpc) in humans] (Hoeffel et al., 2015; McGrath et al., 2015; Ivanovs et al., 2017; Lacaud and Kouskoff, 2017). During this wave, primitive (nucleated) erythroblasts, megakaryocytes, and Mφs are generated. The cells arise as a result of endothelial-to-hematopoietic transition from precursors expressing endothelial markers. The exact cellular source of primitive hematopoietic cells is not yet clear: it has been suggested that this is hemangioblast (HAB), a common precursor of hematopoietic and endothelial cells; however, strong evidence of HAB existence *in vivo* is still missing (Lacaud and Kouskoff, 2017; Yamane, 2018). The emergence of primitive hematopoietic cells directly from mesoderm or HE (i.e., endothelial cells having a potential to generate blood cells) is considered as alternatives (Lacaud and Kouskoff, 2017). Primitive Mφs migrate to the central nervous system to form primitive microglia that can later be partially replaced by definite microglia derived from hematopoietic stem cells (HSCs; Ginhoux et al., 2010; Hoeffel et al., 2015; Ferrero et al., 2018; Hadland and Yoshimoto, 2018). Primitive Mφs also give rise to a small fraction of skin Langerhans cells (Hoeffel et al., 2012; Collin and Milne, 2016). An important characteristic of primitive hematopoiesis is that it is independent on c-Myb transcriptional factor (Tober et al., 2008; Schulz et al., 2012).

The second hematopoietic wave (prodefinitive or the first definitive) also occurs in the YS (E8.25–11.5 in mice; presumably, 28–35 dpc in humans) (Hoeffel et al., 2015; Ivanovs et al., 2017; Lacaud and Kouskoff, 2017; Yamane, 2018). During this wave, HE is formed and generates erythromyeloid progenitors (EMPs) that have erythromyeloid but lack lymphoid potential. EMPs give rise to definitive erythrocytes, megakaryocytes, and myeloid cells (Hoeffel et al., 2015; Lacaud and Kouskoff, 2017; Hadland and Yoshimoto, 2018; Yamane, 2018). EMP-derived Mφs mature in the fetal liver, seed the tissues (other than brain), and form self-renewing TRM pools; their differentiation is c-Myb–independent according to some (Schulz et al., 2012; Dzierzak and Bigas, 2018) but not all (Tober et al., 2008; Frame et al., 2013; Hoeffel et al., 2015) data.

The third (definitive) wave takes place at different sites (i.e., placenta, vitelline, and umbilical arteries), but primarily in the aorta-gonad mesonephros (AGM), where mesoderm-derived HE gives rise to long-term repopulating HSCs and progenitor cells (E10.5–11.5 in mice; around 33 dpc in humans) (Imanirad, 2013; Ivanovs et al., 2014, 2017; Lacaud and Kouskoff, 2017). HSCs migrate to the fetal liver, self-renew, expand, home to the spleen and fetal BM, and initiate adult-type hematopoiesis (Imanirad, 2013; Hoeffel et al., 2015). The third wave is c-Myb–dependent (Lee et al., 2018).

In adults, all blood cells are generated in the BM from HSCs that have a unique capacity to maintain dormancy, self-renew, and enter differentiation (reviewed in detail by Laurenti and Göttgens, 2018).

Overall, three different types of Mφs are generated throughout the lifetime, i.e., primitive, EMP-derived, and HSC-derived. The first two types are HSC-independent.

## General Principles of iMph Differentiation and the Classification of Existing Protocols

### The Main Stages of iMph Differentiation

**FIGURE 2:**
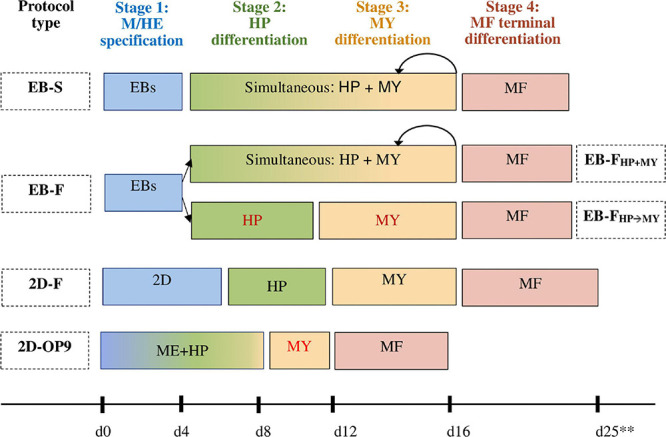
Schematic representation of different protocols used to generate iMphs. In all protocols, the differentiation passes through four main stages: mesoderm commitment and hemogenic endothelium specification (M/HE stage, shown in blue); endothelial-to-hematopoietic transition and the generation of hematopoietic progenitors (HP stage, shown in green); myeloid specification and monocyte formation (MY stage, shown in orange); and terminal differentiation of monocytes into macrophages (MF stage, shown in pink). The protocols differ by the method used to induce M/HE specification and factors added to drive HP and MY stages. In EB-S protocols, iPSCs cultured in low-adhesive conditions form embryoid bodies (EBs); mesoderm is induced within the EBs in the absence of exogenous factors. HP and MY differentiations are driven by the same factors, IL-3 and M-CSF; cells sequentially go through both stages, which cannot be separated from each other. In EB-F protocols, mesoderm is also induced by generating EBs, but its formation is assisted by exogenous factors. HP and MY differentiations are induced either simultaneously by culturing the cells in the presence of IL-3 and M-CSF (EB-F_*HP*__+__*MY*_ protocols) or sequentially by culturing the cells in the presence of hematopoietic factors without M-CSF first (HP stage) and then adding M-CSF to the cultures (MY stage, EB-F_*HP→MY*_ protocols). In 2D-F protocols, mesoderm is induced by culturing iPSCs on matrix-coated plastic in the presence of mesoderm-inducing factors. HP and MY stages are driven sequentially by adding different mixtures of hematopoietic and myeloid-inducing factors. In 2D-OP9 protocols, hematopoietic and myeloid specifications are induced by culturing iPSC on bone marrow (BM) stromal cells. Myeloid progenitors are then expanded in the presence of exogenous factors (GM-CSF). In all protocols, terminal differentiation of iMphs is driven by M-CSF. Rounded arrows show multiple rounds of differentiation. **Because of the differences between the individual protocols, timing is indicated approximately.

The differentiation of iMphs recapitulates many traits of embryonic hematopoiesis. The following four stages of iMph differentiation may be outlined (Figure 2):

(i) mesoderm commitment and HE specification (M/HE stage);
(ii) endothelial-to-hematopoietic transition and the generation of hematopoietic progenitors (HP stage);
(iii) myeloid specification and the formation of monocyte-like cells (MY stage); and
(iv) terminal differentiation of iMphs (MF stage).

The demarcation of the stages is conditional, as several differentiation processes may run simultaneously in the cultures and because in many protocols some of the stages are combined.

Cell transition through the differentiation stages is driven by culture setups, primarily by cytokines, growth factors, and small molecules that are added to the cultures. Individual protocols differ significantly in the combinations of factors that are used and other culture parameters. Based on these conditions, the protocols may be classified into several groups. In this section, we will characterize the main groups of protocols and the principles that they use to direct each differentiation stage. The details of the technical performance of individual protocols and the detailed reference list are provided in *Technical Procedures Used for iMph Differentiation*.

### Two-Dimensional OP9 Stromal Coculture Protocols

Historically, the first differentiations of Mφs from PSCs were achieved by coculturing ESCs with stromal cells that secrete proteins able to promote the proliferation of hematopoietic cells. Several different stromal cell lines have been developed to support hematopoietic differentiation, e.g., bone marrow–derived S17 and OP9 (Kaufman et al., 2001; Vodyanik et al., 2005), YS endothelium cell-derived C166 (Kaufman et al., 2001), AGM-derived UG26, and AM20.1B4 (Ledran et al., 2008; Buckley et al., 2011). Of them, only OP9 has been used for iMph differentiation. OP9 originates from the BM cells of osteopetrosis mice genetically lacking M-CSF. The lack of M-CSF in OP9 cells prevents early monocyte/Mφ bias and supports the generation of various hematopoietic lineages (Lynch et al., 2011). In iMph protocols, PSCs are cocultured on OP9 cell layer until hematopoietic progenitors are generated. MY and MF differentiations are then driven by culturing the cells in the presence of cytokines specific for myeloid differentiation, such as M-CSF and granulocyte-Mφ colony-stimulating factor (GM-CSF) (Choi et al., 2009; Kambal et al., 2011; Senju et al., 2011; Brault et al., 2014). The OP9 coculture system allows generating different types of hematopoietic cells. A fundamental limitation of the method is that the factors secreted by stromal cells and the mechanisms of stromal cell–mediated hematopoietic induction are not fully defined. Additionally, the use of xenogeneic cells reduces the standardizability of the approach and limits its application, considering the clinical focus of current research studies. Therefore, stromal coculture protocols are currently less used for iMph differentiation, and they will not be considered further in the review.

### Classification of the Stromal Cell–Independent Protocols Based on the Performance of the M/HE Stage

**FIGURE 3:**
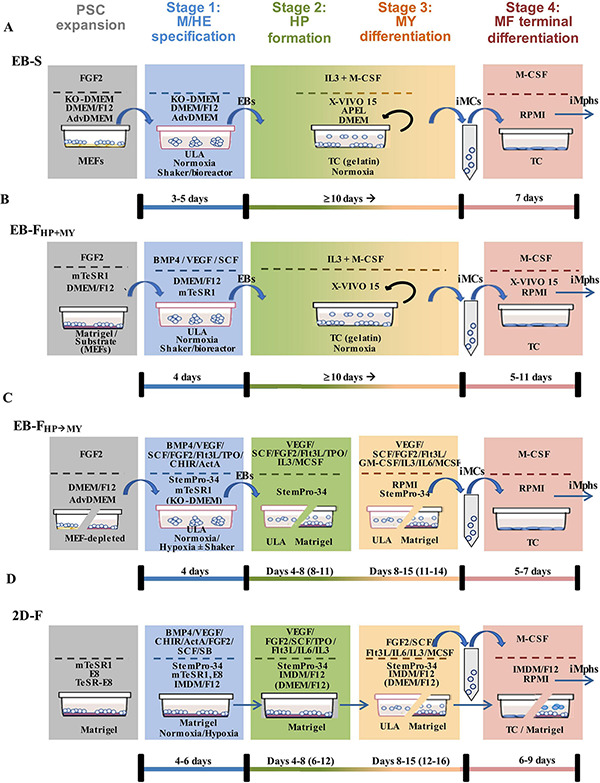
Schematic representation of different types of protocols used to generate iMphs from pluripotent stem cells. Different types of protocols are currently employed to generate iMphs from PSCs **(A)** EB-S protocols. PSCs are expanded on MEFs. At the M/HE stage, mesoderm/HE are induced through the formation of EBs in ULA plastic. For HP and MY stages, EBs are transferred to TC plates and cultured in the presence of IL-3 and M-CSF. Floating cells that appear in the cultures are collected, centrifuged, filtered, and transferred to new TC plates for terminal differentiation (MF stage) in the presence of M-CSF. Remaining cells are restimulated with IL-3 and M-CSF for continuous generation of iMCs. **(B)** EB-F_*HP*__+__*MY*_ protocols. In most protocols, PSCs are expanded on matrix-coated plates. At the M/HE stage, EBs are formed in ULA plastic, where the formation of mesoderm/HE is directed by exogenous factors. For HP and MY stages, EBs are transferred to new TC plates and cultured in the presence of IL-3 and M-CSF. This and further stages are performed exactly as in EB-S protocols. Remaining cells are restimulated with IL-3 and M-CSF for continuous generation of iMCs. **(C)** EB-F_*HP→MY*_ protocols. PSCs are depleted from MEFs prior to differentiation. EBs are formed in ULA plastic, where M/HE stage is directed by exogenous factors in normoxia or hypoxia conditions. After that, EBs are transferred to ULA or Matrigel-coated TC plates, where HP stage is induced by exogenous factors. MY differentiation is directed in the same plates by changing the composition of exogenous factors. Floating cells that appear in the cultures are collected, transferred to TC plates, and terminally differentiated. **(D)** 2D-F protocols. PSCs are always prepared in Matrigel-coated plates in defined media. For M/HE induction, the cells are plated to matrix-coated plates and cultured in the presence of M/HE-inducing exogenous factors in normoxia or hypoxia conditions. HP differentiation is usually induced in the same wells by adding HP-inducing exogenous factors. For MY differentiation, the cells are either transferred to ULA plastic or left in the same Matrigel-coated wells and are stimulated with a new mixture of factors. At the MF stage, floating cells that are formed in ULA conditions are transferred to TC plates and cultured in the presence of M-CSF. If at the MY stage the cells were cultured in Matrigel-coated wells, they continue to be cultured in the same wells; the MF stage is induced by adding M-CSF. In the figure, the lists of factors include all factors that have been used at a given stage by different investigators. More detailed information is provided in Tables 5–7 and Supplementary Table 1. Black curved arrows, continuous rounds of iMC generation. TC, tissue culture plates; MEFs, mouse embryonic fibroblasts; ULA, ultralow-adhesive plates.

The first stage of iMph differentiation starts from PSCs and ends with the formation of mesoderm and HAB/HE cells (Hadland and Yoshimoto, 2018; Lee et al., 2018). Based on the method used to induce M/HE, the protocols may be classified into the following groups (summarized in Figure 2, detailed in Figure 3):

- embryoid body (EB)–based three-dimensional (3D) spontaneous protocols (EB-S);
- EB-based 3D factor-assisted protocols (EB-F); and
- EB-independent two-dimensional (2D) factor–assisted protocols (2D-F).

In EB-S protocols, PSCs are cultured in low-adherent conditions that favor cell aggregation and the formation of EBs. The latter are the multicellular 3D aggregates able to form all three germ layers, including the mesoderm, and to differentiate to diverse populations of adult specialized cells (Itskovitz-Eldor et al., 2000). Cells composing EBs autonomously produce factors and signals required for the differentiation; M/HE are generated spontaneously within the EBs without the addition of exogenous factors (Panicker et al., 2012; van Wilgenburg et al., 2013; Ackermann et al., 2018; see Table 1 for other references).

**TABLE 1:** Variability of iMPh differentiation protocols during iPSC expansion and M/HE stage.

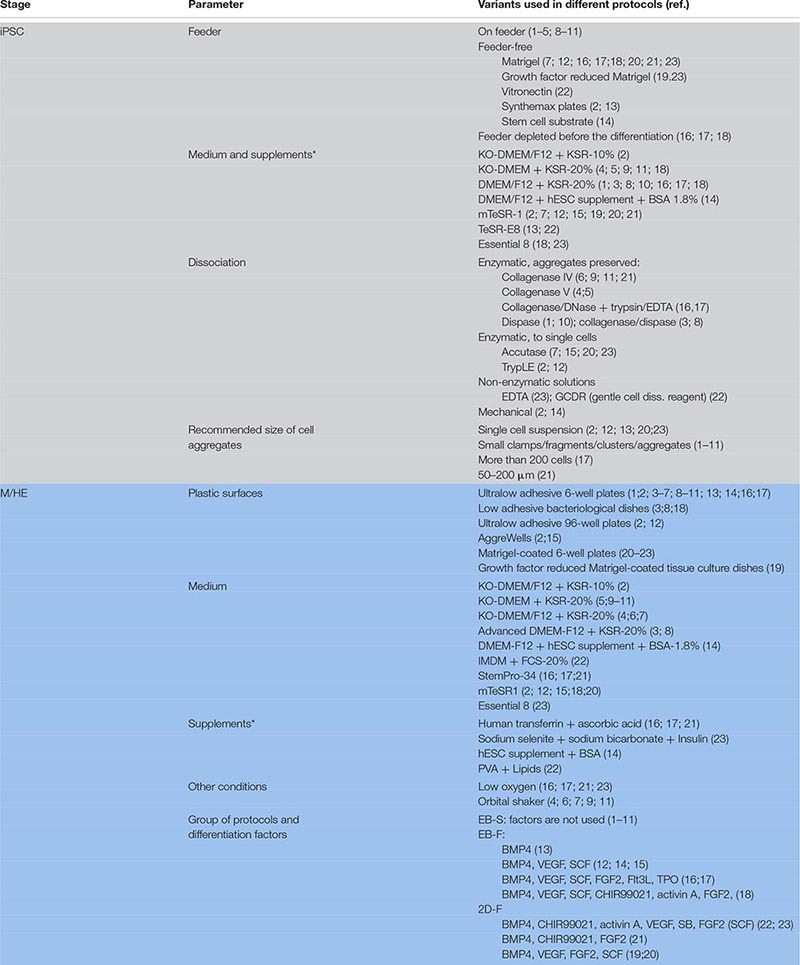

*Standard supplements (NEAA, glutamine/GlutaMAX, β-mercaptoethanol, MTG, penicillin, streptomycin, etc.) are not indicated. For references, see Table 2.

**TABLE 2:** Variability of iMph differentiation protocols at HP, MY, and MF stages.

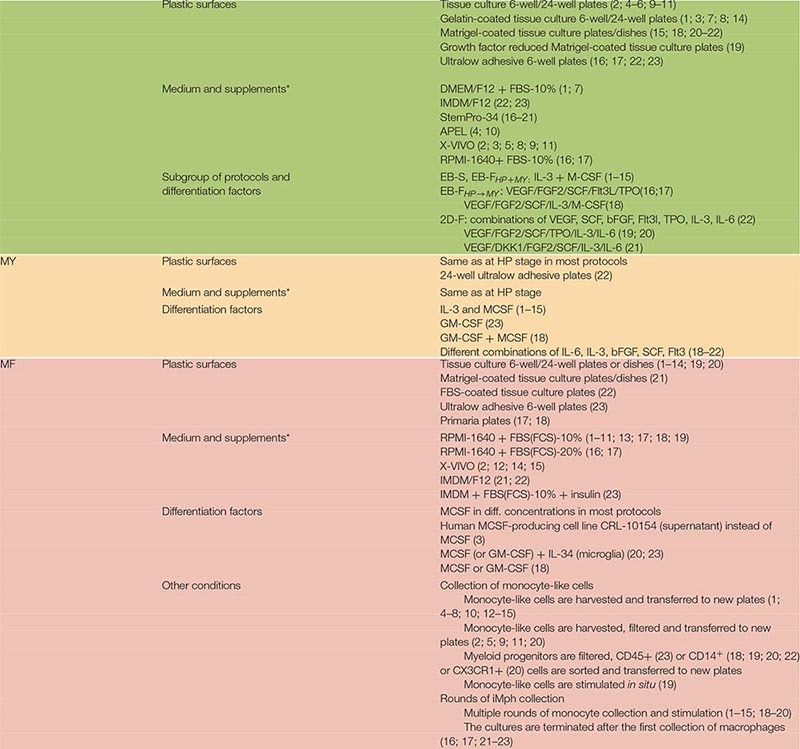

*Standard supplements (NEAA, glutamine/GlutaMAX, β-mercaptoethanol, MTG, penicillin, streptomycin, etc.) are not indicated.

*Protocol list:*

*EB-S protocols (1) Panicker et al., 2012; (2) van Wilgenburg et al., 2013; (3), Alasoo et al., 2015; (4), Lachmann et al., 2015; (5) Neehus et al., 2018; (6); Ackermann et al., 2018; (7) Hong et al., 2018; (8) Mukherjee et al., 2018; (9) Haake et al., 2020; (10) Klatt et al., 2019; (11) Lipus et al., 2020.*

*EB-F_*HP*__+__*MY*_ protocols: (12) Buchrieser et al., 2017; (13), Yeung et al., 2017; (14) Lopez-Yrigoyen et al., 2020; (15) Gutbier et al., 2020.*

*EB-F_*HP*__→__*MY*_: (16) Zhang et al., 2015; (17) Shi et al., 2019; (18) Joshi et al., 2019.*

*2D-F protocols: (19) Yanagimachi et al., 2013; (20) Douvaras et al., 2017; (21) Takata et al., 2017; (22) Cao et al., 2019; (23) Konttinen et al., 2019.*

In EB-F protocols, EBs are also formed, but M/HE specification is assisted by the addition of exogenous M/HE-inducing factors that help to direct the specified trajectory of EB differentiation and increase the efficiency of M/HE formation (van Wilgenburg et al., 2013; Zhang et al., 2015; Buchrieser et al., 2017, see Table 1 for other references). The factors most often used are basic morphogenetic protein 4 (BMP4), vascular endothelial growth factor A (VEGF), and stem cell factor (SCF) (discussed in detail in *Exogenous Factors Used for iMph Differentiation* and *Technical Procedures Used for iMph Differentiation*).

In 2D-F protocols, PSCs are cultured on matrix-coated plates, most often on Matrigel (Yanagimachi et al., 2013; Takata et al., 2017; Cao et al., 2019; Konttinen et al., 2019). The conditions limit 3D diffusion of cells and do not favor the formation of true self-organizing EB structures (Langhans, 2018). As a result, M/HE generation critically depends on exogenous factors; as such, different combinations of BMP-4, VEGF, SCF, fibroblast growth factor 2 (FGF2 or bFGF), Wnt-agonist CHIR99021, and activin A are used (see *Exogenous Factors Used for iMph Differentiation* and *Technical Procedures Used for iMph Differentiation* for details).

### Classification of the Stromal Cell–Independent Protocols Based on the Performance of the HP and MY Stages

After the M/HE stage, HP and MY stages are directed by culturing EBs or on-Matrigel grown cells in the presence of hematopoietic cytokines (Figures 2, 3).

All hematopoietic cytokines may be classified into those that act on multipotent cells [e.g., SCF, interleukin 3 (IL-3), IL-6] and therefore have broad effects on multiple cell lineages and those that are more lineage-specific [e.g., M-CSF or granulocyte colony-stimulating factor (G-CSF)]. The HP differentiation is induced by various combinations of broad-acting cytokines; the MY stage is driven primarily by M-CSF. Based on the list and application timing of hematopoietic cytokines, iMph protocols may be divided into two subgroups.

In the first subgroup, EBs generated during the M/HE stage are cultured in the presence of only two cytokines, IL-3 and M-CSF, which induce HP and MY specifications, respectively. In these conditions, the HP and MY stages are driven simultaneously (“HP+MY” scheme). The approach is used only in EB-based protocols, i.e., in all EB-S and in some EB-F protocols (hereafter referred to as EB-F_*HP*__+__*MY*_).

In the second subgroup, the HP differentiation is driven by a mixture of several broad-acting cytokines, such as VEGF, SCF, Fms-related tyrosine kinase 3 ligand (Flt3L), and so on. The list of cytokines varies between the protocols (discussed in *Technical Procedures Used for iMph Differentiation*). The MY differentiation is driven by M-CSF, which is added either in the presence of a reducing number of broad-acting hematopoietic cytokines or alone. This leads the cells along the following path: HE → hematopoietic progenitors → monocyte-like cells, i.e., HP and MY differentiations go on sequentially, and the stages can be separated, although conditionally. The “HP → MY” scheme is applied in some EB-F (EB-F_*HP*__→__*MY*_) and all 2D-F protocols.

As a result of the HP/MY stages, floating round-shaped cells exhibiting the main characteristics of monocytes (i.e., the general morphology, the expression of CD14, and the ability to differentiate into Mφs) appear in the cultures and are referred to as “monocytes” or, better, monocyte-like cells (in this review referred to as iMCs).

At the MF stage, iMCs are subjected to terminal differentiation by cultivating them in the presence of M-CSF. The variations include the use of different M-CSF concentrations and additional cytokines (discussed in *Technical Procedures Used for iMph Differentiation*).

The technical procedures and the peculiarities of individual protocols are discussed in *Technical Procedures Used for iMph Differentiation* after we review the main biological activities of the factors used for iMph differentiation.

## Exogenous Factors Used for iMph Differentiation

Exogenous factors play a pivotal role in driving iMph differentiation. This section summarizes the main characteristics of the factors used, as this is important for understanding the principles and the variability of iMph generation protocols. Summarized information on all factors is also presented in Tables 3, 4.

**TABLE 3:** Exogenous factors and small molecules used during M/HE stage of iMPh differentiation.

**Factor**	**Receptor(s)**	**Main signal transducers**	**Hematopoiesis-related biological activities**	**Stage when used**	**Type of the protocol**	**Main references**
BMP4	BMPR1, BMPR2 STKRs	Smad1/5/8 p38MAPK, JNK	Multiple developmental processes including the formation of mesoderm and hemogenic endothelium	M/HE	All EB-F All 2D-F	Boxman et al., 2016; Hong et al., 2018; Malaguti et al., 2013; Nostro et al., 2008; Pauklin and Vallier, 2015; Sharma et al., 2017; Wang et al., 2014
FGF2	FGFR1 FGFR2 FGFR3 FGFR4 RTKs	JAK/STAT RAS/RAF/MAPK PI3K/AKT PLC-γ	Maintains pluripotency, cell proliferation, survival, differentiation; is involved in embryonic development and tissue repair; in conjunction with other factors exhibits mesoderm-inducing activity and supports hemangioblast-like cells. Inhibits BMP4	PSC expansion M/HE HP	All Some of EB-F, All 2D-F All EB-F_HP→MY_, most 2D-F All EB-F_HP→MY_, most 2D-F	Mossahebi-Mohammadi et al., 2020 Thisse and Thisse, 2005; Tiong et al., 2013
CHIR99021		GSK3 inhibitor; Wnt agonist	Inhibits GSK3, increases Wnt-signaling. Wnt: induces the formation of primitive streak and mesoderm; maintains self-renewal and pluripotency of ESCs; induces iPSC differentiation to vascular progenitors and definitive hematopoietic cells; in embryogenesis, is involved in multiple developmental processes	M/HE	Some EB-F_HP→MY_, Some 2D-F	Boxman et al., 2016; Cao et al., 2019; Davidson et al., 2012; Galat et al., 2017; Lindsley et al., 2006; Moon, 2005; Nostro et al., 2008; Sturgeon et al., 2014
Activin A	ACVR1 ACVR2 STKRs	Smad2/3 Smad4 (p38 MAPK, ERK1/2, JNK)	Promotes endoderm induction; in the presence of SCF/Flt3l stimulates hematopoietic-fated mesoderm, promotes hematopoietic progenitor expansion	M/HE	Some EB-F_HP→MY_, Some 2D-F	Cerdan et al., 2012 Kubo et al., 2004 Pauklin and Vallier, 2015; Tsuchida et al., 2009
VEGFA	VEGFR2 (KDR)	PLC-γ PI3K/AKT p38 MAPK FAK/paxilline NCK SFKs	Vascular development, hemangioblast formation, expansion of committed hematopoietic progenitors	M/HE HP	All EB-F, All 2D-F All EB-F_HP→MY_, Most 2D-F	Abhinand et al., 2016 Damert et al., 2002; Goldie et al., 2008 Kennedy et al., 2007; Koch and Claesson-Welsh, 2012; Park et al., 2004; Pearson et al., 2008

*ACVR, activin A receptor; AGM, aorta-gonad mesonephros; EHT- endothelial-to-hematogenic transition; FAK, focal adhesion kinase; FIMP, Fms-interacting protein; FL, fetal liver; GSK3, glycogen synthase kinase 3; JAK, Janus kinase; MAPK, mitogen-activated protein kinase; NSK, nonspecific serine/threonine protein kinase; PI3K, phosphoinositide 3-kinases; AKT, protein kinase B; PKs, protein kinases; PLC-γ, phospholipase C; RAS, Ras proteins; RAF, Raf proteins; ERK, extracellular signal-regulated kinases; RTK, receptor tyrosine kinases; SFK, Src family of protein tyrosine kinases; SMAD, transforming growth factor-β superfamily member signals; STAT, signal transducer and activator of transcription; STKRs, serine/threonine kinase receptors; VEGFRs, receptors for vascular endothelial growth factor.*

**TABLE 4:** Exogenous factors used during HP and MY stages of iMph differentiation.

**Factor**	**Receptor(s)**	**Main signal transducers**	**Hematopoiesis-related biological activities**	**Stage when used**	**Type of the protocol**	**Main references**
SCF	c-kit (CD117) RTKIII	PI3K, RAS/RAF/ERK1/2 JAK/STAT PLC-γ SFKs	Promotes cell survival, proliferation, differentiation and migration; survival and expansion of HSPCs in the BM; survival of AGM and FL HSCs; formation of YS EMPs (microglia is SCF-independent) Combined with IL-3, IL-6, and/or TPO, promotes basal proliferation of progenitor cells; in the presence of lineage-specific cytokines assists HSPC differentiation	M/HE HP	All EB-F, some 2D-F All EB-F_HP→MY_ most 2D-F	Azzoni et al., 2018 Kent et al., 2008; Kimura et al., 2011; Rönnstrand, 2004; Rybtsov et al., 2014
Flt3l	Flt3 (CD135) RTKIII	RAS/RAF/ERK1/2 PI3K STAT/STAT5a	Promotes proliferation of HSCs and progenitor cells, particularly of granulomonocytic lineage Synergizes with SCF and TPO in the induction of CD34+ cell expansion; in combination with M-CSF, GM-CSF, and G-CSF promotes the formation of myeloid colonies	M/HE HP	Some EB-F_HP→MY_ All EB-F_HP→MY_, some 2D-F	Gabbianelli et al., 1995; Gilliland and Griffin, 2002; Kikushige et al., 2008; McKenna et al., 1995; Sonoda et al., 1997; Tsapogas et al., 2017; Xiao et al., 1999; Wodnar-Filipowicz, 2003
TPO	Mpl	JAK/STAT PI3K/AKT RAS/RAF/ERK1/2	Promotes megakaryocyte differentiation, HSC survival and quiescence; HSC self-renewal and expansion in posttransplantation conditions; HSC expansion in FL and *in vitro*	HP	Some EB-F_HP→MY_, Some 2D-F	Decker et al., 2018; de Graaf and Metcalf, 2011; Kaushansky, 2005; Saka et al., 2018; Sasazawa et al., 2015; Yoshihara et al., 2007
IL-6	IL-6R/gp130	JAK/STAT3 (PI3K/AKT MEK/ERK)	Multiplication of HSPCs and promotion of myeloid differentiation	HP MY	Some 2D-F Some 2D-F	Lokau et al., 2017; Wolf et al., 2014; Rose-John, 2018; Zegeye et al., 2018
IL-3	IL-3Rα/ IL-3Rβ	JAK/STAT, Ras/Raf/ERK PI3K/AKT	Supports the proliferation and the differentiation of HSCs, early myeloid progenitors and B lymphocytes. In embryogenesis promotes EHT, the emergence and the survival/proliferation of HSCs in AGM, YS, and placenta	HP MY	Almost all Almost all	Ackermann et al., 2020; Bertrand et al., 2010; He et al., 2010; Mui et al., 1995; Robin et al., 2006; Rybtsov et al., 2011; Torigoe et al., 1992; Quelle et al., 1994
M-CSF	CSFR1 RTKIII	PI3K/AKT, PLC, ERK1/2 SFK-ERK5 FIMP	Supports hematopoietic progenitor cell proliferation; monocytes/macrophage differentiation, activation, mobilization, stimulation of phagocytosis and M2-like bias	MY MF	All All	Stanley and Chitu, 2014; Jones and Ricardo, 2013; Sherr, 1990; Jack et al., 2009

*For abbreviations, see Table 3.*

### BMP4

Basic morphogenetic protein 4 is a multifunctional protein that belongs to the transforming growth factor-β (TGF-β) superfamily. The factor acts by binding to BMPR1 and BMPR2 receptors that activate canonical (Smad1/Smad5/Smad8–dependent) and non-canonical [p38–mitogen-activated protein kinase (MAPK) and PI3K/AKT mediated] signaling pathways (Wang et al., 2014). During embryogenesis, BMP4-triggered pathways are implicated in multiple differentiation processes, including the induction of mesoderm and the formation of HE (Nostro et al., 2008; Malaguti et al., 2013; Pauklin and Vallier, 2015; Boxman et al., 2016; Sharma et al., 2017). *In vitro*, BMP4 effects depend on the dose and the duration of treatment. A low BMP4 concentration supports cell pluripotency, whereas higher doses prime mesoderm differentiation (Malaguti et al., 2013). Short-term exposure of cells to BMP4 (24–72 h) induces mesoderm (Zhang P. et al., 2008; Boxman et al., 2016; Naticchia et al., 2018), whereas long-term treatment (7 days) promotes mesoderm differentiation into trophoblast (Xu et al., 2002).

For iMph differentiation, BMP4 is used in all factor-dependent protocols to promote M/HE specification (Table 5).

**TABLE 5:** Combinations of exogenous factors used to drive iPSC differentiation at the M/HE stage.

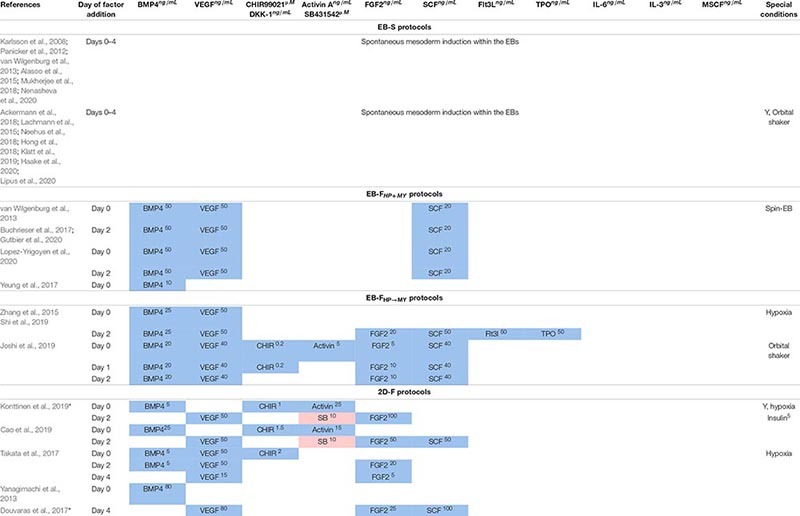

Color clues: blue, M/HE stage; pink, inhibitors. Y, Y-27632. *Microglia differentiation protocols.

### FGF2

Fibroblast growth factor 2 belongs to the FGF protein family that includes 22 ligands and four tyrosine kinase receptors. It signals through RAS/RAF/MAPK, PI3K/AKT, phospholipase C γ (PLC-γ), and Janus kinase (JAK)/signal transducer and activator of transcription (STAT)–mediated pathways. FGF2 supports cell pluripotent state, and it is also involved in the regulation of cell survival, proliferation, differentiation, embryonic development, and tissue repair (Thisse and Thisse, 2005; Tiong et al., 2013; Mossahebi-Mohammadi et al., 2020).

Concerning hematopoietic differentiation, FGF2 was shown to be involved in the formation of hematopoietic-fated mesoderm in amphibians, but not in humans (Cerdan et al., 2012). FGF2 is in complex interactions with other mesoderm-inducing pathways. Particularly, it forms a positive regulatory loop with a mesoderm-specific T-box transcriptional factor Brachyury (Schulte-Merker and Smith, 1995; Papaioannou, 2014), but there is a negative regulatory loop between FGF2 and BMP4 (Naticchia et al., 2018; Schliermann and Nickel, 2018). High levels of FGF2 inhibited primitive blood differentiation and promoted endothelial cell fate (Nakazawa et al., 2006). Yet, in conjunction with other factors, FGF2 can induce mesoderm activity and support the induction of HAB-like cells and cell proliferation/survival (Takata et al., 2017).

In iMph differentiation protocols, FGF2 is used at predifferentiation stage to support iPSC pluripotency during their expansion and to assist M/HE and MY stages (Table 5). Because of FGF2 capacity to maintain cell pluripotency and antagonize BMP4, many investigators pay special attention to exclude FGF2 from culture medium during the first 2 to 3 days of iPSC differentiation (Table 5) or even 3 to 5 days prior to the start of iPSC differentiation (Ackermann et al., 2018). However, some authors did add FGF2 to cell cultures at differentiation day 0, either in reduced concentrations (EB-S protocol, Lachmann et al., 2015) or in combination with BMP4 (EB-F protocol, Joshi et al., 2019). A few EB-F protocols did not use FGF2 at all (Xu et al., 2012; Buchrieser et al., 2017; Lopez-Yrigoyen et al., 2020).

### VEGF

Vascular endothelial growth factor A is a member of a family of proteins that also includes VEGF-B, VEGF-C, VEGF-D, and placental growth factor. VEGF signals through the receptor VEGFR2 (also called KDR and Flk1) that, in embryogenesis, is expressed by mesodermal, angioblast, and endothelial cells. The signaling cascades include PI3K/AKT, Ras/Raf/MAPK, PLC-γ, and FAK/paxillin (reviewed by Koch and Claesson-Welsh, 2012). VEGF is secreted by the endoderm and plays multiple roles in developmental processes; particularly, it is mandatory for vascular development, contributes to the formation of HAB, and is necessary for the expansion and the differentiation of committed hematopoietic progenitors (Shalaby et al., 1995; Ferrara et al., 1996; Damert et al., 2002; Park et al., 2004; Kennedy et al., 2007; Goldie et al., 2008; Pearson et al., 2008).

Vascular endothelial growth factor A is used in most factor-dependent protocols during the M/HE stage and in most 2D-F and EB-F_*HP*__→__*MY*_ protocols during the HP stage (Table 5).

### CHIR99021

CHIR99021 is the inhibitor of Gsk3β and the activator of canonical and non-canonical Wnt signaling pathways (Moon, 2005). The pathways are involved in many developmental processes (i.e., body axis specification, germ line formation, organogenesis), including the formation of primitive streak and mesoderm (Huelsken et al., 2000; Kelly et al., 2004; Nostro et al., 2008; Davidson et al., 2012). *In vitro*, CHIR99021 accelerates the onset of primitive streak/mesoderm and promotes the generation of HE capable of definitive hematopoiesis (Boxman et al., 2016; Galat et al., 2017). A natural inhibitor of Wnt signaling, Dickkopf-related protein 1 (DKK1), impairs mesoderm generation (Lindsley et al., 2006). It was demonstrated that the *in vitro* formation of mesoderm from PSCs requires the Wnt pathway to be unaltered between days 1.5 and 2.5 of cell differentiation (Lindsley et al., 2006; Boxman et al., 2016). Accordingly, in iMph protocols, CHIR99021 is added to PSCs on differentiation days 0 to 2; it has been used in some EB-F and 2D-F protocols (Table 5).

### Activin/Nodal

Activin and Nodal are members of the TGF-β superfamily of morphogens; both signal through the same serine/threonine-protein kinase receptors ACVR1 and ACVR2 and activate canonical Smad2/Smad3 mediated and non-canonical (p38-MAPK, ERK1/2, and JNK mediated) signaling pathways (Tsuchida et al., 2009). In human ESC studies, activin/Nodal were shown to either maintain pluripotency or induce endoderm and to be in antagonistic relationships with BMP4 (Pauklin and Vallier, 2015). However, in the presence of BMP4 and hematopoietic cytokines, such as SCF and Flt3L, activin A promoted the formation of Brachuyry^+^ hematopoietic-fated mesoderm (Kubo et al., 2004; Cerdan et al., 2012). Activin A contributes to hematopoiesis also by promoting the expansion of hematopoietic progenitor cells (Cerdan et al., 2012). The hematopoietic effects of activin A and Wnt differ: activin A supports the generation of primitive progenitors and KDR^+^CD235a^+^ HAB cells, whereas Wnt/β-catenin signaling favors the generation of definitive KDR^+^CD235a^–^ progenitors (Sturgeon et al., 2014).

Activin A has been used in some factor-dependent protocols during M/HE stage in combination with CHIR99021 and BMP4 (Table 5). Whether activin A is prerequisite or surplus for M/HE generation and how it interacts with BMP4 and Wnt-mediated signaling during the initial differentiation stages remains to be elucidated.

### SCF

Stem cell factor is a broad-acting hematopoietic cytokine that acts at the early stages of hematopoietic differentiation, both during embryogenesis and in adults. SCF receptor, c-kit (or CD117), is expressed on hematopoietic stem and progenitor cells (HSPCs), mast cells, and also on a variety of other cells not related to hematopoiesis. SCF activates PI3K, RAS/RAF/ERK, JAK/STAT, Src and PLC-γ and regulates the apoptosis, proliferation, differentiation, and migration of c-kit receptor–expressing cells (Rönnstrand, 2004).

During embryogenesis, SCF drives the generation of YS EMPs and the survival of HSCs in AGM and fetal liver (Kimura et al., 2011). Of note, microglia that originate from primitive Mφs are SCF independent (Rybtsov et al., 2014; Azzoni et al., 2018). In adults, SCF is produced in the BM HSC niche and supports the proliferation and the survival of HSPCs (Kent et al., 2008). *In vitro* and in combination with other broad-acting cytokines [i.e., IL-3, IL-6, and/or trombopoietin (TPO)], SCF stimulates progenitor cell proliferation; in the presence of lineage-specific cytokines, it assists HSPC differentiation (Ahmed, 2020).

Stem cell factor is used in most factor-dependent protocols during the M/HE stage and in all factor-dependent protocols, during the HP stage (Tables 5, 6).

**TABLE 6:** Combinations of exogenous factors used to drive iMph differentiation at HP and MY stages.

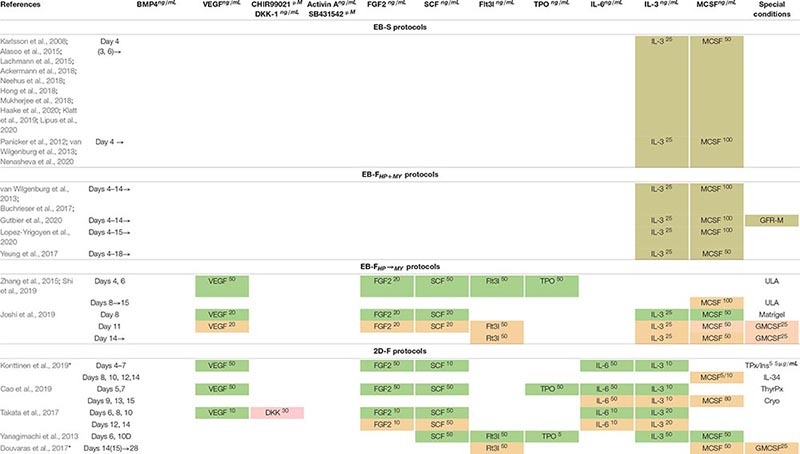

*Color clues: green, HP stage; orange, MY stage; khaki, HP and MY stages induced simultaneously (“HP+MY” scheme”); pink, inhibitor. Cryo, cryopreservation; EPO, erythropoietin; ULA, ultralow adhesive plastic; GFR-M, growth factor–reduced Matrigel-coated plates; TPx, thyroid peroxidase; Ins, insulin.*

### Flt3L

Fms-related tyrosine kinase 3 ligand, another broad-acting hematopoietic cytokine, is produced by BM fibroblasts and T lymphocytes. Flt3L binds to the Flt3 receptor (CD135) that in humans is expressed on HSCs, common myeloid, granulocyte/Mφ, and early lymphoid progenitors (Xiao et al., 1999; Kikushige et al., 2008). Flt3 ligation activates RAS/RAF/ERK, PI3K, and STAT3/STAT5 signaling pathways and induces the proliferation of Flt3-expressing cells, primarily, those of granulomonocytic lineage (McKenna et al., 1995; Sonoda et al., 1997; McKenna et al., 2000; Gilliland and Griffin, 2002; Wodnar-Filipowicz, 2003; Tsapogas et al., 2017). *In vitro*, Flt3L has a limited effect when used alone, but it synergizes with other cytokines. Particularly, in combination with SCF and TPO, it induced the expansion of cord blood CD34^+^ cells; in conjunction with M-CSF, GM-CSF, and G-CSF, it stimulated myelopoiesis (Gabbianelli et al., 1995; Gilliland and Griffin, 2002; Wodnar-Filipowicz, 2003).

For iMph differentiation, Flt3L has been used in some factor-dependent protocols in combination with other hematopoietic cytokines mostly during HP and MY stages (Tables 5, 6).

### TPO

Trombopoietin, a glycoprotein hormone, is produced in many organs, primarily in the liver, kidney, and BM. Ligation of TPO activates JAK/STAT–, PI3K/AKT–, and RAS/RAF/ERK–mediated signaling pathways (Kaushansky, 2005; Sasazawa et al., 2015; Saka et al., 2018). TPO receptor, Mpl, is expressed by megakaryocytes, platelets, HSCs, and HAB (de Graaf and Metcalf, 2011). Accordingly, TPO supports megakaryocyte differentiation and maintains HSC survival; it also has promyelocytic effect. In steady-state conditions, TPO supports HSC quiescence (Alexander et al., 1996; Ballmaier et al., 2003; Yoshihara et al., 2007; de Graaf and Metcalf, 2011; Decker et al., 2018); in posttransplantation conditions, it induces HSC self-renewal and expansion (Fox et al., 2002; Soares-da-Silva et al., 2020). During embryogenesis, TPO was shown to support the survival and the expansion of HSCs in mouse fetal liver (Petit-Cocault et al., 2007); its role in YS hematopoiesis is less clear. *In vitro*, TPO supports megakaryocyte progenitors and promotes the survival and the proliferation of BM HSPCs; the effects are enhanced in the presence of Flt3L and SCF (Ramsfjell et al., 1996; Borge et al., 1997; Zhang et al., 2018). Forced expression of TPO in human ESCs had promegakaryocytic and promyeloid effects (de Graaf and Metcalf, 2011; Soares-da-Silva et al., 2020).

For iMph generation, TPO has been used in combination with Flt3L and/or SCF in a few EB-F and 2D-F protocols at the M/HE and HP stages (Tables 5, 6).

### IL-6

Interleukin 6 is a member of the IL-6 cytokine family; it is produced primarily by innate immune (monocytes/Mφs) and stromal (fibroblasts) cells, as well as by different types of endothelial and epithelial cells. The IL-6 receptor is composed of two subunits, IL-6R (that recognizes specifically IL-6) and gp130 (this is responsible for signal transduction and is common to all IL-6 family cytokines) (Lokau et al., 2017). Gp130 is expressed on all cells, whereas IL-6R is expressed on hepatocytes and certain subpopulations of leukocytes. IL-6 can act on cells that do not express IL-6R through the trans-signaling mechanism, which involves the cleavage of IL-6R from IL-6R–expressing cells in the presence of IL-6, the formation of IL-6–sIL-6R complex, and its interaction with the membrane gp130 (Wolf et al., 2014; Lokau et al., 2017; Rose-John, 2018). IL-6 signaling is mediated via JAK/STAT3; trans-signaling also activates the PI3K/AKT and the MEK/ERK pathways (Zegeye et al., 2018).

Interleukin 6 is a pleiotropic cytokine involved in the development and the regulation of inflammation and immune response. Regarding hematopoiesis, the main IL-6 activities are the multiplication of HSPCs and the promotion of myeloid differentiation, both *in vivo* (Bernad et al., 1994) and *in vitro* (Reynaud et al., 2011; Mirantes et al., 2014; Schürch et al., 2014). In the context of inflammation, IL-6 induces emergency granulopoiesis, even in the absence of GM-CSF and G-CSF (Ishihara and Hirano, 2002; Maeda et al., 2009). During zebrafish embryogenesis, IL-6 promoted the generation of hematopoietic cells and HSCs (Tie et al., 2019). IL-6 has been used in some 2D-F protocols at the HP and MY stages (Table 6).

### IL-3

Interleukin 3 is a 20- to 32-kDa glycoprotein produced predominantly by activated T lymphocytes and to a lesser extent by other cells including myeloid cells. The IL-3 receptor consists of the IL-3–specific IL-3Rα chain and the β chain common for IL-3, IL-5, and GM-CSF receptors (Kitamura et al., 1991). IL-3R is expressed by HSCs, myeloid cells, and B lymphocytes; its ligation activates JAK/STAT, RAS/RAF/ERK, and PI3K/AKT signaling pathways and multiple tyrosine kinases, i.e., LYN, FYN, SRC, SYK, TEC1, and HCK (Torigoe et al., 1992; Quelle et al., 1994; Mui et al., 1995; Reddy et al., 2000; Chang et al., 2003).

In adults, IL-3 supports the proliferation and the differentiation of HSCs, early myeloid progenitors, and B lymphocytes (Bujko et al., 2019). During embryogenesis, the role of IL-3 has long been attributed to its capacity to stimulate the proliferation and the differentiation of mesodermal progenitors (Bertrand et al., 2010; He et al., 2010) and to promote the emergence and/or the survival/proliferation of HSCs located in the AGM, YS, and placenta (Robin et al., 2006; Rybtsov et al., 2011). Recently, Ackermann et al. (2020), using an *in vitro* human “hemanoid model,” have demonstrated that (i) IL-3 is required for endothelial-to-hematopoietic transition; (ii) this IL-3 function cannot be replaced by SCF; (iii) IL-3 is sufficient for the continuous production of immature myeloid progenitors in the *in vitro* iMph differentiation model (Ackermann et al., 2020). Thus, IL-3 can both induce hematopoietic progenitors and stimulate their myeloid differentiation, which explains its unique role in iMph differentiation protocols: it is used in all protocols at the HP/MY stages (Table 6).

### M-CSF

Mφ colony-stimulating factor is a lineage-specific hematopoietic factor essential for the differentiation, survival, and functioning of mononuclear phagocytes, including monocyte/Mφs, dendritic cells, and osteoclasts (reviewed in Jones and Ricardo, 2013; Stanley and Chitu, 2014; Mun et al., 2020). M-CSF is produced by mesenchymal and epithelial cells located in different tissues (Ryan et al., 2001). M-CSF receptor (CSFR1 or CD115) is a tyrosine kinase receptor encoded by the c-fms proto-oncogene. M-CSF receptor is expressed at low levels on HSCs and at higher levels on monocytes and tissue Mφs. Its ligation activates PI3K/AKT, Src, PLC-γ, and ERK kinases and SHP-1 phosphatase, promoting cell survival, proliferation, and differentiation (Sherr, 1990; Jack et al., 2009; Stanley and Chitu, 2014). At the level of hematopoietic progenitor cells, M-CSF drives cell proliferation and instructs myeloid-fate changes (Mossadegh-Keller et al., 2013; Jin and Kruth, 2016). Acting on monocytes, it promotes cell survival, mobilization, and the differentiation to Mφs. In Mφs, M-CSF activates phagocytosis and skews cell activity to an anti-inflammatory tissue-repair type (Svensson et al., 2011; Jones and Ricardo, 2013). Lack of M-CSF results in a severe deficiency in tissue Mφs accompanied by multiple developmental abnormalities (Wiktor-Jedrzejczak et al., 1990; Pollard and Stanley, 1996; Jones and Ricardo, 2013). Of note, M-CSF and IL-3 may synergize in inducing monopoiesis: IL-3 enhances the expression of M-CSF receptor; M-CSF induces transcriptional factor c-Fos that enhances IL-3 driven monopoiesis (Jack et al., 2009; Sheng et al., 2014). M-CSF is a key cytokine for the generation of iMphs and their precursors (Tables 6, 7).

**TABLE 7:** Combinations of exogenous factors used at MF stage (terminal iMph differentiation).

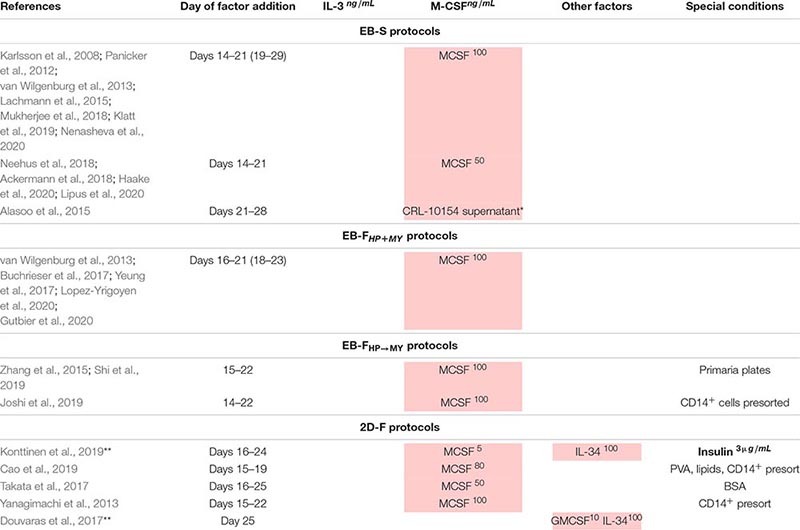

Color clues: pink, MF stage. *MCSF-producing cell line. **Microglia differentiation protocols.

## Technical Procedures Used for iMph Differentiation

### Preparation of PSCs for iMph Differentiation

#### Cell Sources

Historically, human iMphs were first differentiated from ESCs (Karlsson et al., 2008; Klimchenko et al., 2011), and ESCs are still used for iMph generation (van Wilgenburg et al., 2013; Yanagimachi et al., 2013; Douvaras et al., 2017; Hong et al., 2018). However, because of ethical constraints, poor availability, and the impossibility of obtaining human ESCs with any given genetic background, currently iPSCs are used more often. iPSCs are most often obtained from commercially available cell lines, or they are prepared in-house from dermal fibroblasts (Panicker et al., 2012; Buchrieser et al., 2017; Konttinen et al., 2019; Lopez-Yrigoyen et al., 2019; Haake et al., 2020), peripheral blood monocytes (Zhang et al., 2015; Joshi et al., 2019), mobilized peripheral blood or BM CD34^+^ cells (Lachmann et al., 2014; Ackermann et al., 2018; Cao et al., 2019; Haake et al., 2020). iMphs were also generated from iPSCs derived from kidney epithelium cells and peripheral blood erythroblasts (Zhang et al., 2015; Cao et al., 2019).

#### PSC Expansion: Feeder-Dependent and Feeder-Free Conditions

Before the differentiation starts, PSCs need to be expanded. There are two major types of PSC expansion protocols, feeder-dependent and feeder-free (Table 1, Supplementary Table 1). In feeder-dependent protocols, PSCs are grown on feeder cells, for which mitotically inactivated mouse embryo fibroblasts (MEFs) are most commonly used. MEFs produce extracellular matrix and factors supporting cell stemness, and their use is a cheap and easy way to expand and maintain PSCs in an undifferentiated state (reviewed in detail by Yu et al., 2015). However, different lots of feeder cells may differ, which reduces the reproducibility of the experiments. More importantly, the use of animal-derived cells poses a risk of inducing immune reactions and transferring zoonotic pathogens, and it is restricted in clinically oriented studies. Human-derived feeder cells allow avoiding using the xeno-system, but retain the risk of infection, and they are not fully defined or reproducible.

To avoid the limitations, feeder-free culture systems have been developed, in which cells are grown on commercial surfaces covered with growth factor–reduced Matrigel (Yanagimachi et al., 2013) or recombinant xeno-free extracellular matrices Vitronectin (Cao et al., 2019), Cellstart^TM^ substrate (Lopez-Yrigoyen et al., 2020), or Synthemax^TM^ (van Wilgenburg et al., 2013; see Supplementary Table 1 for details). More often, plastic surfaces are coated with matrices in-house, e.g., with Matrigel (Buchrieser et al., 2017; Takata et al., 2017; Hong et al., 2018; Konttinen et al., 2019; Shi et al., 2019) or vitronectin (Mukherjee et al., 2018; Cao et al., 2019). Because of the high cost of xeno-free surfaces and matrices, intermediate types of protocols have been developed, in which PSCs are expanded on MEFs but are depleted from feeder cells before the differentiation; feeder depletion is achieved by a 2-day culture on Matrigel-coated plates (Zhang et al., 2015; Takata et al., 2017; Cao et al., 2019; Konttinen et al., 2019; Shi et al., 2019).

If growing on feeder cells, PSCs are usually expanded in a basal medium, most often in knockout (KO)–Dulbecco modified eagle medium (DMEM) or DMEM/F12 supplemented with Knockout^TM^ Serum Replacement (KSR), a defined serum-free formulate, and other additives. In feeder-free conditions, media formulated specifically to support human ESC/iPSCs in feeder-free conditions are used; these include mTeSR1^TM^ (mTESR), mTESR^TM^-E8^TM^ (mTESR-E8; both from Stem Cell Technologies), or Essential 8^TM^ (E8, Thermo Fischer Scientific; Table 1, Supplementary Table 1). To inhibit cell differentiation and support cell pluripotency, FGF2 is always added to the medium, which is changed daily to compensate for rapid FGF2 degradation.

#### PSC Dissociation and Harvesting

Pluripotent stem cells are cultured until they reach 80 to 90% confluency (Zhang et al., 2015; Takata et al., 2017; Ackermann et al., 2018), at which point they are passaged and cultured further until they expand to a number of wells, needed for iMph differentiation. Recommendations regarding the optimal number of PSC passages are contradictory, e.g., “beyond 20 passages” (Zhang et al., 2015) or “kept to minimum” (Buchrieser et al., 2017).

Harvesting PSCs for iMph differentiation is a critical procedure that influences the efficacy of the experiments. PSCs are adhesive cells that grow in colonies. To start the differentiation, the cells need to be detached from the surfaces while preserving cell viability and differentiation capacity. Three main methods are used for of PSC collection, i.e., enzymatic digestion, non-enzymatic dissociation, and mechanical harvesting (Table 1, Supplementary Table 1). For the enzymatic digestion, the cells are treated with collagenase, dispase, or their combinations. These enzymes preserve cell clumps, which supports cell viability. Some authors, however, prefer to use trypsin-like enzyme (TripLE) or accutase that disrupt PSC colonies to single cells. The approach helps to obtain uniform EBs, which is important for their further synchronous and efficient differentiation (Pettinato et al., 2015). However, single-cell PSCs have poor survival and an increased risk of abnormal karyotypes (Beers et al., 2012). Therefore, in the protocols where PSCs are disrupted to a single-cell suspension, the cells are immediately forced to aggregate, e.g., by plating PSCs into round-bottom 96-well plates and centrifuging the plates at 100–500*g* immediately after the plating (van Wilgenburg et al., 2013; Buchrieser et al., 2017). When PSCs are disrupted to single cells during their harvesting, the inhibitors of the rho-associated kinase (ROCK) pathway (e.g., Y-27632 or Thiazovivin/Tzv) are added for 24 h (van Wilgenburg et al., 2013; Buchrieser et al., 2017; Hong et al., 2018) or even 48 h (Konttinen et al., 2019) of culture. Some authors use ROCK inhibitors even when passaging or harvesting PSCs in aggregates (Lachmann et al., 2015; Ackermann et al., 2018; Konttinen et al., 2019; Lipus et al., 2020). Regardless of the enzyme used for PSC dissociation, a key step is to inactivate and/or dilute enzymes sufficiently to prevent reduced cell attachment and ensure cell survival in subsequent cultures.

As a way of non-enzymatic dissociation, EDTA treatment was suggested. The approach is cheap and easy to do, and EDTA-treated PSCs were shown to be long-lived, preserve karyotype, and have a high survival efficiency (Beers et al., 2012). Yet, in iMph differentiation protocols, EDTA treatment is rarely used (Mukherjee et al., 2018; Konttinen et al., 2019), which may be attributed to a somewhat lower efficiency of cell disaggregation, variable adhesion of different PSC lines, and/or traditional preferences for enzymatic cell disruption.

Non-enzymatic dissociation of cells can also be performed using commercially available Gentle Cell Dissociation Reagent (GCDR, Stem Cell Technologies), an enzyme- and animal component-free solution that does not require washing/centrifugation after the treatment (used by Cao et al., 2019). GCDR allows dissociating cells into clumps or single cells depending on the goals of the study (determined by treatment duration and temperature).

In the mechanical approach, PSC colonies are lifted from feeder cell or matrix-coated surfaces using cell scraper (van Wilgenburg et al., 2013) or a special cell passaging tool, e.g., StemPro EZPassage Disposable Stem Cell Passaging Tool (Lopez-Yrigoyen et al., 2020). Both approaches require manual experience. The latter cuts cell colonies into pieces of uniform size increasing the reproducibility of EB generation, but it is more expensive.

The size of PSC aggregates is a method-specific parameter that affects cell differentiation efficacy. It is generally agreed that large aggregates are preferred as they support cell viability (Beers et al., 2012; Shi et al., 2019). And yet, cells located within large aggregates may be less accessible for external differentiation factors. The exact size of PSC aggregates, considered to be optimal, differs among the protocols and can be more than 200 cells (Sturgeon et al., 2014; Shi et al., 2019), 10 to 20 cells (Grigoriadis et al., 2010) or no more than 6 to 10 cells (Dege and Sturgeon, 2017). As mentioned above, some authors prefer to start the differentiation with a single-cell suspension to support EB uniformity (van Wilgenburg et al., 2013; Buchrieser et al., 2017).

After PSCs are collected, they are put into cultures to start M/HE specification.

### EB-S Protocols

In EB-S protocols, M/HE specification is induced through the formation of EBs (general schemes are presented in Figure 3; the details of the protocols are summarized in Tables 5–7 and Supplementary Table 1).

Pluripotent stem cells are most often prepared on MEFs and then are cultured in low-adhesion conditions that favor cell aggregation, proximity and 3D communications. These include cell culture in (i) ultralow-adhesive or bacterial-grade plates/dishes (Lachmann et al., 2014; Alasoo et al., 2015; Zhang et al., 2015; Mukherjee et al., 2018); (ii) hanging drops (Foty, 2011); and (iii) low-adhesive round-bottom 96-well plates (van Wilgenburg et al., 2013). Other conditions favoring uniform PSC aggregation and EB formation include a quick spin of iPSC-containing plates before the start of cell culture (“spin-EBs”; van Wilgenburg et al., 2013; Buchrieser et al., 2017) and stirring cultured cells using an orbital shaker (Lachmann et al., 2015; Neehus et al., 2018; Joshi et al., 2019; Haake et al., 2020) or bioreactor (Ackermann et al., 2018). The cultures are generally maintained in basal media, such as DMEM/F12, advanced DMEM/F12, or KO-DMEM supplemented with Knockout^TM^ serum replacement and other additives. The appearance of mesoderm is marked by the expression of *brachyury* and KDR; HAB/HE-like cells are detected based on the coexpression of KDR, endothelial (CD144, CD31), and early hematopoietic (CD34) markers and the lack of the expression of CD45 and CD73 (Lachmann et al., 2015; Buchrieser et al., 2017; Cao et al., 2019; Shi et al., 2019; Ackermann et al., 2020). M/HE generation usually takes 4 to 5 days. Variations include 3 days (Alasoo et al., 2015) and 8 to 11 days (Hong et al., 2018).

For HP and MY differentiations, EBs are manually transferred to tissue culture (TC) plates/dishes that some authors coat with gelatin (Panicker et al., 2012; Alasoo et al., 2015; Mukherjee et al., 2018). The cells are cultured in the presence of IL-3 and M-CSF in a serum-free X-VIVO 15 medium (Lonza; most protocols), serum-free and animal component-free STEMdiff^TM^APEL^TM^ medium (APEL, Stem Cell Technologies; Lachmann et al., 2015; Klatt et al., 2019) or supplemented DMEM (Panicker et al., 2012; Hong et al., 2018). The medium is changed every 3 to 7 days. Suspensive iMCs appear in the cultures around differentiation days 15 to 20 and are collected for terminal differentiation. The remaining adherent cells are fed with a new IL-3/M-CSF containing medium to induce the next round of iMC generation; the latter may last for several months, and iMCs are harvested once or twice a week over the course of several months (Panicker et al., 2012; Lachmann et al., 2015; Ackermann et al., 2018) or even up to a year (van Wilgenburg et al., 2013; Table 8). For the MF stage, gathered iMCs are filtered through 70- to 100-μm mesh filters and transferred into new TC plates, where they are cultured in a supplemented RPMI-1640 medium (most protocols) or X-VIVO 15 medium (van Wilgenburg et al., 2013; Nenasheva et al., 2020) containing M-CSF. Variations include the addition of IL-3 (Hong et al., 2018) or the use of the supernatant from M-CSF producing CRL-10154 cell line (Alasoo et al., 2015). iMphs mature, on average, in 5 to 7 days; they are collected for the analyses as they are (“M0” Mφs) or are polarized using interferon γ (IFN-γ)/lipopolysaccharide (LPS) or IL-4 prior to the collection. The variations among the protocols largely include the details of PSC preparation, the use of orbital shaker at the M/HE stage, the medium utilized at the M/HE and HP+MY stages, and concentrations of M-CSF (Table 7; Supplementary Table 1).

**TABLE 8:** Advantages and limitations of the main groups of iMph differentiation protocols.

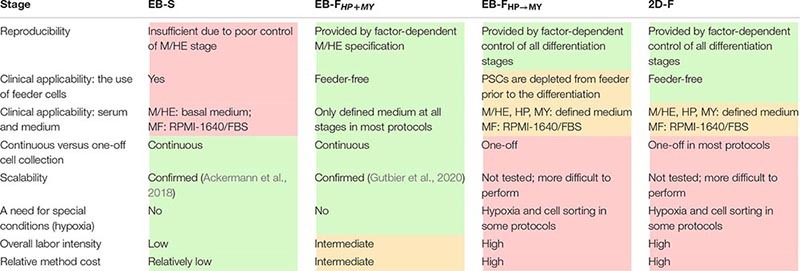

*Green, advantages; pink, disadvantages; yellow, intermediate.*

### EB-F Protocols

In most EB-F protocols, PSCs are expanded on Matrigel or are depleted from feeder prior to the differentiation. The main difference from EB-S protocols is that M/HE specification is assisted by exogenous factors; the HP and MY stages are performed using “HP+MY” or “HP→MY” schemes (summarized in Figure 3; details provided in Tables 5–7 and Supplementary Table 1).

In EB-F_HP+MY_ protocols, EBs were generated by culturing PSCs in low-adhesive conditions in mTeSR1 or supplemented DMEM/F12 medium containing BMP4, VEGF, and SCF (van Wilgenburg et al., 2013; Buchrieser et al., 2017; Lopez-Yrigoyen et al., 2020) or BMP4 only (Yeung et al., 2017). On day 4, EBs were transferred to TC plates, and the HP+MY and MF stages were induced exactly as they are in EB-S protocols. Briefly, iMCs were generated in X-VIVO 15 medium containing IL-3 and M-CSF; iMph terminal differentiation was induced by M-CSF in new TC plates and supplemented RPMI-1640.

EB-F_HP→MY_ protocols are more complex and variable. Zhang et al. (2015) and Shi et al. (2019) generated EBs in hypoxia conditions in low-adhesive plates and StemPro^TM^ 34 medium (StemPro-34; Thermo Fisher Scientific; developed specifically to support hematopoietic cells). The medium was supplemented with BMP4 and VEGF; on day 2, cytokine mixture was complemented with FGF2, SCF, Flt3L, and TPO. For HP differentiation, EBs were cultured in the same conditions, except for the exclusion of BMP4 from the culture medium (days 4–8). At the MY stage, StemPro-34 was replaced by supplemented RPMI-1640 containing M-CSF. For iMph differentiation, iMCs were transferred to Corning^®^ Primaria^TM^ Culture Plates and stimulated with M-CSF in supplemented RPMI-1640 medium. The main features of these two studies are that (i) during the M/HE and HP stages, the cells were cultured in hypoxia conditions; (ii) the M/HE, HP and MY stages were all run in low-adhesive plastic. It is worth noting that StemPro-34 was supplemented with MTG, ascorbic acid, and human transferrin (as it is done in all the other protocols where this medium is used).

Joshi et al. (2019) used even more complex combinations of factors. They started the M/HE stage by culturing iPSCs in mTeSR1 medium in the presence of BMP4, VEGF, CHIR99021, activin A, FGF2, and SCF and proceeded by excluding activin A and CHIR99021 on days 1 and 2, respectively. On day 8 (HP differentiation), EBs were transferred to Matrigel-coated plates and stimulated with VEGF, SCF, FGF2, IL-3, and M-CSF. On day 11, this cytokine mixture was supplemented with Flt3L and GM-CSF; on day 14, VEGF, FGF2, and SCF were excluded, and the cells were cultured in the presence of Flt3L, IL-3, M-CSF, and GM-CSF. Floating cells that appeared in the cultures were magnetically sorted to isolate the CD14^+^ population, which was used for terminal differentiation performed in supplemented RPMI-1640 medium in the presence of M-CSF.

To summarize, in EB-F protocols, M/HE specification is primarily driven by BMP4, VEGF, and SCF, which may be complemented with other factors. CHIR99021 and activin A, key mesoderm inducers, are not generally used in EB-F protocols, apparently because Wnt- and activin-mediated signaling may be provided endogenously within the EBs. FGF2 is usually added to the cultures not earlier than day 2, which is due to its capacity to maintain cell pluripotency and antagonize BMP4. However, Joshi et al. (2019) did include CHIR99021, activin A, and FGF2 in their complex cytokine mixture used to drive the M/HE stage starting day 0. It remains unclear whether the addition of these factors was critical for M/HE and iMph generation or whether they were surplus, given the successful generation of EBs and iMphs in other EB-based protocols that utilized lower quantities of factors. An important point to note is that besides the differences discussed above, the protocols also differ in the concentrations in which exogenous factors are added, e.g., BMP4, 10 to 50 ng/mL; FGF2, 5 to 20 ng/mL; SCF, 20 to 50 ng/mL; M to CSF, 50 to 100 ng/mL (Tables 5–7).

### 2D-F Protocols

Two-dimensional factor protocols are aimed at the generation of iMphs in defined feeder-free conditions. This is a heterogeneous group of protocols, in which the differentiation is driven by complex mixtures of factors that differ among the protocols, and so do many other culture conditions, such as medium, plastic ware, and several procedures (Figure 3, Tables 5–7, and Supplementary Table 1).

Takata et al. (2017) cultured cells in Matrigel-coated plates throughout all differentiation stages. The M/HE stage was induced by BMP4, CHIR99021, and VEGF in StemPro 34 medium. For the formation of HAB, on day 2 CHIR99021 was substituted for FGF2. On day 4, BMP4 was omitted, and the cells were cultured in the presence of VEGF and FGF2 only. For HP commitment, VEGF, FGF2, SCF, IL-6, IL-3, and DKK-1 were used (days 6–12). At the MY stage, VEGF and DKK-1 were excluded, and hematopoietic progenitors were matured in the presence of FGF2, SCF, IL-3, and IL-6 (days 12–16). On day 16, the medium was refreshed with supplemented IMDM medium containing M-CSF, which induced iMph terminal differentiation. The latter were collected once on day 25. During days 0–8, the cells were cultured in hypoxia conditions.

Cao et al. (2019) also used BMP4 and CHIR99021 to induce M/HE. However, they did not add VEGF at the beginning of cell differentiation, but added activin A. The differentiation was performed in IF9S (supplemented IMDM) medium. On day 2, BMP4 was excluded, CHIR99021 and activin A were substituted for FGF2 and activin A inhibitor SB431543, and VEGF and SCF were added. For HP differentiation, VEGF, FGF2, and SCF were complemented with IL-6, IL-3, and TPO. Before the MY stage, the cells were dissociated and transferred to ULA plates where they were cultured in suspension in the presence of IL-6, IL-3, and M-CSF. Generated CD14^+^ iMCs were magnetically sorted and cryopreserved. For terminal differentiation, cryopreserved iMCs were thawed and differentiated in TC plates coated with fetal calf serum in a supplemented IMDM/F12 medium containing M-CSF.

In contrast to the previous two studies, Yanagimachi et al. (2013) did not use CHIR99021 and activin A for iMph differentiation; M/HE specification was induced by high concentrations of BMP4 (80 ng/mL; growth factor-reduced Matrigel coated plates; mTeSR1 medium). On day 4, mTeSR1 was replaced by StemPro-34 containing VEGF, FGF2, and SCF. The generation of HP progenitors was driven by SCF, Flt3L, TPO, IL-3, and M-CSF cocktail; the MY stage was driven by Flt3L, M-CSF, and GM-CSF. On days 15 to 28, CD14^+^ was positively sorted and terminally differentiated in a supplemented RPMI-1640 medium containing M-CSF.

A high heterogeneity of culture conditions, primarily of factors used for iMph differentiation, suggests that some of the factors may not be necessary and that optimal conditions for iMph generation are yet to be determined.

### Advantages and Limitations of Different iMph Differentiation Protocols

The diversity of iMph differentiation protocols raises questions on their advantages and limitations (Table 8).

Embryoid body spontaneous protocols are cheap and easy to do; they support prolonged iMC generation, which provides investigators with a continuous source of cells for the experimentation and increases the cumulative iMph yield. However, EB-S protocols have limited reproducibility, largely because the differentiation success depends on parameters that are difficult to control (i.e., the size and the homogeneity of EBs, the efficacy of M/HE formation). Additionally, most EB-S protocols are feeder-dependent and utilize a chemically undefined medium, which limits their future clinical applications.

EB-F_HP→MY_ and 2D-F protocols have the advantage of using exogenous factors to sequentially drive and control all differentiation stages. Additionally, the protocols use feeder-free or feeder-depleted conditions and chemically defined serum-free medium (except for the MF stage; Table 8; Supplementary Table 1). The price for these advantages is that protocols are more expensive and labor-intensive. A further drawback is the one-off collection of iMphs. In this regard, it is worth noting that cryopreservation and the accumulation of independent batches of iMCs have recently been suggested (Cao et al., 2019); the approach has a potential to be broadly used in the field to compensate the limitations of one-off collection protocols.

EB-F_HP + MY_ protocols combine the main advantages of all other protocols. Specifically, the M/HE stage is factor-controlled; HP and MY stages are driven by only two factors, which reduces labor intensity and cost; all stages are run in a defined medium and feeder-free conditions and enable continuous iMph generation (Table 8, Supplementary Table 1).

The yield of iMphs obtained in different protocols is an important point to consider. However, reports on the comparative yields of iMCs/iMphs generated by the same group using the same PSC line(s) but different protocols are missing. Making comparisons between the protocols employed by different groups is difficult, as the protocols vary in PSC lines, culture conditions, the duration of iMph generation, and the method used to calculate the iMC/iMph yield (e.g., per well or per starting PSC numbers/wells; Table 9). When we converted reported data to estimate the yield per well of a 6-well plate (assuming that the growth area is 9.5 cm^2^ and that the well volume is 3 mL), we found that the highest yields were obtained in EB-F_HP + MY_ protocols (Table 9; Gutbier et al., 2020; Lopez-Yrigoyen et al., 2020), especially in the protocol by Gutbier et al. (2020) specially designed for a large-scale production of iMphs.

**TABLE 9:** The yield of iMCs/iMphs obtained in different types of protocols.

References	**Yield description from the manuscript**	Calculated yield of iMCs (per well in a 6-well plate)*	
		**Per week**	**Cumulative**

**EB-S protocols**			
Panicker et al., 2012	Continuous monocyte production starting weeks 2–3; monocytes were harvested every 4–5 days; under optimal conditions, more than 2 million cells were harvested per week from four to five EBs (4–10 EBs/well of 6-well plate)	**∼ 2 × 10^6^/well**	NA
van Wilgenburg et al., 2013	≥1 × 10^7^ cells from a 6-well plate; collected weekly; production continued for up to 1 year; the cumulative yield was ∼10^7^ per plate over 3 months	0.13 × 10^6^/well	∼1.7 × 10^6^ over 3 months
Lachmann et al., 2015	0.5–1 × 10^6^ cells/well/week during 2 months, up to 4–5 months	0.5–1 × 10^6^	8–16 × 10^6^ for 4 months
Ackermann et al., 2018	250 mL bioreactor: a stable production of ∼1–3 × 10^7^ iMphs per week starting week 3; maintained for more than 5 weeks	∼0.12–0.36 × 10^6^	0.6–1.8 × 10^6^
Mukherjee et al., 2018	Harvested every 4–5 days for 6–8 months after which precursor number dropped significantly	NA	NA

**EB-F_HP+MY_ protocols**			

Buchrieser et al., 2017	Over a period of 30 days, an average of 3 × 10^6^ monocytes/macrophages were collected per well	∼0.75 × 10^6^	∼3 × 10^6^
Lopez-Yrigoyen et al., 2020	On average, 2.59 × 10^6^ ± 0.54 cells were harvested from a 6 well plate on days 16–28; after day 28, an average of 4.64 × 10^6^ ± 0.94 of suspension cells per 6 well plate were harvested; from day 80 onward, the number of cells started to drop; cells were harvested every 3–4 days	∼0.86 × 10^6^ (days 16–28) **∼1.55** × **10^6^ (days 28–80)**	**∼ 1.3** × **10^7^**
Gutbier et al., 2020	2D 1,000-cm^2^ cultures: series of 18–25 harvests with single harvest yields of up to 6 × 10^8^ cells from 2D 1,000-cm^2^ cultures	**5.7** × **10^6^**	**∼1**–**1.4** × **10^8^**

**EB-F_HP→MY_ protocols**			

Shi et al., 2019	Up to 2 × 10^7^ cells per 6-well plate of iPSCs within 24 days	NA	NA (one-off collection)
Zhang et al., 2015	Up to 2 × 10^7^ of CD45^+^/CD18^+^ differentiated macrophages per 6-well plate of confluent iPSCs	NA	NA (one-off collection)

**2D-F protocols**			

Cao et al., 2019	~5 × 10^6^ of CD14^+^ cells from each 6-well plate of hiPSCs (one-off collection).	∼0.8 × 10^6^	∼0.8 × 10^6^
Takata et al., 2017	10–20 cells per starting primary stem cell	NA	NA
Yanagimachi et al., 2013	1.3 × 10^6^ ± 0.3 × 10^6^ cells per 100 mm culture dish at each medium exchange (medium changed on days 15–28 every 3–4 days)	0.22 × 10^6^	0.88 × 10^6^

*In different studies, the yield of suspensive cells/iMphs is calculated in different ways. Where possible, the data were converted to estimate the yield per well on a 6-well plate (assuming that the approximate growth area is 9.5 cm^2^ and that the well volume is 3 mL). The highest yields are highlighted in bold.

## Phenotypic and Functional Characteristics of the Resultsing iMphs

Despite the variability of iMph differentiation protocols, all of them result in the generation of cells that exhibit similar morphological, phenotypic, and functional properties. In all studies, iMphs appeared large, highly vacuolated, and equipped with pseudopodia cells expressing typical Mφ markers, i.e., CD45, CD11b, and CD14. The evaluation of the expression of other markers demonstrated iMph expression of CD16, CD64, CD68, CD80, CD86, CD163, CD206, CD195, CD192, CX3CR1, CD115, and HLA-DR, although the list of markers that were analyzed and the levels of their expression differed between the studies (Panicker et al., 2014; Lachmann et al., 2015; Zhang et al., 2015; Ackermann et al., 2018; Mukherjee et al., 2018). Of note, several studies reported low-level expression of HLA-DR and CD16 (van Wilgenburg et al., 2013; Mukherjee et al., 2018) and the coexpression of CD80/CD86 and CD163/CD206 by iMphs (Lopez-Yrigoyen et al., 2020), which altogether allowed characterizing iMphs as a low-polarized “naive-like” population (Nenasheva et al., 2020). Phagocytic activity, an indicator of Mφ nature, was assessed in almost all iMph studies and was always high. iMphs were infectable with intracellular bacteria and were able to restrict the growth of *Salmonella typhi*, *Salmonella typhimurium*, *Pseudomonas aeruginosa*, and *Mycobacterium tuberculosis* (Hale et al., 2015; Ackermann et al., 2018; Haake et al., 2020; Nenasheva et al., 2020). Following the infection with *Chlamydia trachomatis*, iMphs supported the full infectious life cycle of the pathogen, mimicking the infection of MDMs (Yeung et al., 2017).

Induced pluripotent stem cell response to inflammatory stimuli was in the focus of the analysis in many studies. The cells were polarizable and responded to LPS/IFN-γ by characteristic changes in their phenotype, transcriptomic, and secretory profiles (Alasoo et al., 2015; Zhang et al., 2015). Some authors used IFN-γ stimulation not only to study iMph reactivity, but also as an additional step of iMph differentiation/priming. This resulted, in particular, in the upregulated expression of MHC molecules and effective antigen presentation (Joshi et al., 2019). iMph responses to IL-4 and IL-10 were also registered, although some authors reported poor iMph reactivity to IL-4, supposedly due to an initial M2 bias of iMphs (Zhang et al., 2015).

Many studies reported phenotypic, functional, and transcriptomic similarities between iMphs and MDMs (Alasoo et al., 2015; Zhang et al., 2015; Yeung et al., 2017; Mukherjee et al., 2018). However, stable differences between the populations were also identified. These included a higher expression of extracellular matrix and fibroblast genes (i.e., *PDGFRA*, *PDGFRB*, *LOX*, *FGF1*, *TIMP1*, *COL11A1*, *COL3A1*, *COL1A1*, etc.) and a lower expression of genes associated with immune response (i.e., *CCL5*, *CXCL9*, *CXCL10*, and MHC class II molecules) by iMphs (Alasoo et al., 2015; Zhang et al., 2015).

Thus, iMphs generated in different protocols exhibit similar general Mφ characteristics and are reminiscent of MDMs. At the same time, iMph fine characteristics differ from MDMs, and it was suggested that iMphs recapitulate embryonic-origin TRMs rather than MDMs (Buchrieser et al., 2017; Takata et al., 2017; Lee et al., 2018). To date, the similarity between iMphs and TRMs has not been studied in detail, and we do not know to what extent iMphs generated using different protocols are similar.

## Markers of iMph Differentiation and iMph Origin

The trajectories of hematopoietic differentiation following iMph generation pose an intriguing question. These were followed only in a few studies and using various combinations of markers. The findings can be summarized as follows. Mesodermal KDR^+^CD144^+^CD34^–^ cells appear in the cultures by day 4 (reported for 2D-F protocols, Cao et al., 2019; Konttinen et al., 2019). Cells coexpressing endothelial and hematopoietic markers (i.e., KDR^+^CD34^+^ or CD144^+^CD34^+^CD73^–^) and classified as HAB or HE emerge by day 6 (2D-F protocols, Yanagimachi et al., 2013; Cao et al., 2019). Early hematopoietic CD43^+^CD34^+^ progenitors are detected around day 8 (EB-F protocol, Zhang et al., 2015). The majority of CD34^+^CD43^+^ progenitors express CD235a and CD41a and exhibit erythromegakaryocyte potential; a small proportion of CD43^+^ cells are CD235a^–^CD41a^–^CD45^+^, and these have myeloid potential. At late differentiation stages, the expressions of CD235a and CD41a are lost, and the percentage of CD45^+^ cells gradually increases (2D-F protocol, Cao et al., 2019).

It is generally assumed that *in vitro* hematopoietic differentiation of PSCs, including the generation of iMphs, resembles primitive rather than definitive hematopoiesis (Vanhee et al., 2015; Shi et al., 2019). In the case of iMphs, this notion is supported by the appearance of CD235^+^/CD41^+^ cells at the early differentiation stages (shown in EB-F and 2D-F protocols, Zhang et al., 2015; Konttinen et al., 2019) and by the possibility of generating iMphs in the absence of c-Myb (Buchrieser et al., 2017). However, the formation of CD235^+^CD41^+^ HAB-like cells does not exclude the possibility of generating “early definitive” Mφs in the same cultures. Also, the independence of iMphs from c-Myb was shown in EB-F_*HP*__+__*MY*_ protocol, in which only IL-3 and M-CSF were used for HP and MY differentiation (Buchrieser et al., 2017). Other types of protocols utilize many other factors that are involved in definitive hematopoiesis, such as SCF, Flt3L, and IL-6. Further, several factor-dependent protocols used CHIR99021, an agonist of Wnt signaling, which was shown to bias the hematopoiesis toward a definitive type (Sturgeon et al., 2014). Finally, the generation of EMPs during iMph differentiation was directly documented (Cao et al., 2019; Konttinen et al., 2019). Thus, primitive and early definitive iMphs are likely to be coproduced in the cultures, the ratio between them is not known, and it may depend on the protocol used for iMph differentiation.

Both primitive and EMP-derived Mφs are HSC-independent. Whether HSC-dependent Mφs can be generated from iPSCs is another important question. Several studies reported the generation of multipotent definitive progenitors in iPSC cultures. Kennedy et al. (2012) observed the formation of CD43^–/*low*^ expandable definitive hematopoietic progenitors having lymphoid potential when iPSCs were cultured in the presence of stromal cells and activin A inhibitor. Vanhee et al. (2015) detected the generation of definitive CD34^+^CD43^+^CD45^–/lo^ cells at the late stages of EB-OP9 cocultures. Although the cells had restricted granulocytic hematopoietic potential, they expressed c-Myc, a sign of definitive hematopoiesis. Dege and Sturgeon (2017) described the generation of erythromyelolymphoid multilineage definitive progenitors in iPSC cultures directed using EB-F–like protocol. Thus, definitive-like multilineage progenitors can be generated from iPSCs *ex vivo*. At present, we do not know to what extent these cells contribute to the iMph pool. However, it is clear that the cells do not possess the self-renewal and reconstitution potential characteristic of HSCs and that they are preferentially generated in the prolonged PSC-OP9 cocultures (Garcia-Alegria et al., 2018).

Besides the analysis of the early stages of iMph differentiation, the characterization of later stages is of interest. In particular, it will be interesting to know whether the pathways of the MY stage differ among the protocols that exploit “HP+MY” and “HP→MY” differentiation schemes. The precursors of iMphs are suspension cells that appear at the end of the MY stage, exhibit general common characteristics with blood monocytes (van Wilgenburg et al., 2013), and are usually classified as monocytes (iMCs in this review). Direct comparison of iMCs and blood monocytes revealed some morphological and phenotypic differences, such as larger vesicles, a larger diameter, a higher expression of CD163, and a lower expression of CD16 and CD86 on iMCs (van Wilgenburg et al., 2013; Nenasheva et al., 2020). More importantly, iMCs and blood monocytes differ fundamentally by their origin (i.e., they are HSC-independent and HSC-dependent, respectively). Thus, an open question is whether iMCs can be categorized as monocytes. More in-depth analyses are needed to understand to what extent iMCs and blood monocytes, i.e., cells originating from different progenitors through different pathways, converge.

Overall, there are currently not very many studies addressing iMph differentiation trajectories, and no study has compared the trajectories of iMph differentiation using different protocols. Knowing the differentiation pathways and the properties of iMCs/iMphs obtained in different protocols is all the more important given that iMphs have multiple promising applications in the future.

## iMph Applications and Prospects

There are several promising application areas where iMphs have the potential to be used.

### Disease Modeling

Gene mutations and an impaired phagocyte function underlie several rare hereditary diseases. For all of them, iMphs represent a unique model for studying the fundamental mechanisms of disease pathogenesis and searching for therapeutic molecular targets. Two main approaches are used to create iMph-based disease models: (i) generating iMphs from patient-derived iPSCs and (ii) introducing disease-associated mutations to iPSCs derived from healthy donors followed by the generation of iPSC-derived iMphs. The first approach has successfully been used to model Gaucher disease, Tangier disease, familial Mediterranean fever, chronic granulomatous disease (CGD), early onset sarcoidosis, Alzheimer disease, Parkinson disease, and others (Panicker et al., 2012; Brault et al., 2014; Zhang et al., 2015; Aflaki et al., 2016; Haenseler et al., 2017; Takata et al., 2017; Brownjohn et al., 2018; Takada et al., 2018; Shiba et al., 2019; Mukhopadhyay et al., 2020). In the second approach, the introduction of p47-ΔGT mutation allowed to model CGD (Klatt et al., 2019), and iMphs bearing genetic KOs of IL-10RA, IL-10RB, STAT1, or STAT3 modeled the very-early onset bowel disease (VEOBD) (Mukhopadhyay et al., 2020; Sens et al., 2020).

### Modeling Mφ–Pathogen Interactions

Macrophages play a pivotal role in pathogen clearance. The fact that iMphs are infectable with various intracellular bacteria (e.g., *S. typhimurium*, *P. aeruginosa*, *M. tuberculosis*) and viruses (e.g., HIV, ZIKA, and dengue) allows using them as a standardized model to study Mφ-pathogen interactions and to search for key targets to reinforce a Mφ-mediated immune defense (van Wilgenburg et al., 2013; Hale et al., 2015; Ackermann et al., 2018; Lang et al., 2018; Bernard et al., 2020; Haake et al., 2020; Nenasheva et al., 2020; O’Keeffe et al., 2020).

### Developing iMph-Based Cell Therapy

Although it is understood that clinical use of iPSC-derived cells has limitations, several new directions in iMph-based cell therapy are being developed (reviewed in Zhang and Reilly, 2017), including the attempts to improve the safety of the approach (Lipus et al., 2020). The proof of principle comes from *in vitro* and experimental *in vivo* studies that have demonstrated the possibility of correcting genetic mutations and improving phagocyte functions using the iMph approach. In the aforementioned iMph models of CGD and VEOBD, CRSPR/Cas9 gene therapy restored hampered iMph functions *in vitro* (Klatt et al., 2019; Sens et al., 2020).

CSFR2b^–/–^ mice model pulmonary alveolar proteinosis (PAP), a severe hereditary respiratory disease in humans. Pulmonary transplantation of gene-edited host Mφs resulted in a long-term engraftment and a beneficial therapeutic effect in mice (Mucci et al., 2016). In another study, human iMphs engrafted, differentiated to alveolar Mφs, and reduced PAP in humanized PAP mice (Happle et al., 2018).

Using a model of acute *P. aeruginosa* infection in immunodeficient hIL-3/GM-CSF-KI mice with impaired alveolar Mφ development, Ackermann et al. (2018) demonstrated that it is possible to enhance pulmonary immunity by an intratracheal injection of human iMphs at the moment of the infection.

Induced pluripotent stem cells expressing a single-chain antibody specific to amyloid β or CD20 exhibited efficient antibody-specific phagocytosis of amyloid β and B-cell leukemia cells (Senju et al., 2011).

In the tissues, TRMs interact with and shape tissue-specific cells through the secretion of soluble mediators and direct cell–cell contacts. It was suggested that iMphs may be used *in vitro* to create the necessary microenvironment and facilitate the development of other cells destined for regenerative medicine [e.g., for bone regeneration (Jeon et al., 2016)].

Recently, methods of generating proliferating iPSC/ESC-derived myeloid cell lines resembling iMphs were suggested; when being genetically modified to express IFNI, these cells inhibited disseminated gastric and colon cancer and melanoma in experimental studies (Koba et al., 2013; Haga et al., 2014; Miyashita et al., 2016).

### Drug Testing

The usefulness of iMphs as a new platform for therapeutic development was demonstrated in the models of Gaucher disease, Parkinson disease, and *Leishmania* infection (Panicker et al., 2014; Aflaki et al., 2016; O’Keeffe et al., 2020). Han et al. (2019) used the advantage that iMphs represent a homogeneous population and utilized them to screen a 3,716-compound library for their activity against intracellular *M. tuberculosis.* The approach allowed identifying a new compound active against both extracellular and intracellular *M. tuberculosis.*

### Other Applications

Because iMphs represent a homogeneous, standardizable, and genetically editable population, they provide a unique opportunity to study Mφ biology, including the role of specific individual genes in cell functionality, like it was recently done by several groups (Zhang et al., 2017; Hall-Roberts et al., 2020; Navarro-Guerrero et al., 2021).

## Discussion

The generation of iMphs from iPSCs is a recently developed technique that enjoys increasing interest. So far, several different approaches to generating iMphs have been elaborated. In all of them, the Mφ nature of iMphs was confirmed by characteristic cell morphology, phenotype, and functionality, including phagocytic and chemotactic activity, infectability, and responsiveness to inflammatory stimuli. A general similarity between iMphs and MDMs at the transcriptional level was also demonstrated. This created a basis for the development of various iMph applications, including disease modeling, drug testing, and cell-based therapy. The advantages of the iMphs model include the possibility of modeling human TRMs, as well as generating genetically identical and editable Mφ populations and to potentially scaling the cell generation technique. Despite the rapid progress in the field, several fundamental and technical outstanding questions remain.

### iMph Origin and Comparison With Other Monocyte/Mφ Populations

It is assumed that iMphs model TRMs. This primarily emphasizes the HSC-independent origin of both cell populations. However, during embryogenesis, there are two HSC-independent waves, the first (primitive) and the second (early definitive). TRMs develop as a result of the second wave; Mφs generated during the first wave give rise primarily to microglia and a small fraction of skin Langerhans cells (Ginhoux et al., 2010; Hoeffel et al., 2015; Collin and Milne, 2016). As discussed in this review, most authors agree that iMph differentiation models primitive hematopoiesis (Buchrieser et al., 2017; Lee et al., 2018). The key questions are as follows: (i) do iMphs, indeed, differentiate exclusively via the primitive-like pathway? (ii) If so, to what extent do iMphs model TRMs? (iii) If not, should we refine iMph differentiation pathways? Of note, data showing the formation of EMPs at the early stages of iMph differentiation support their “early definitive” origin.

Another question related to iMph identity is whether and to what degree iMphs and their floating precursors generated at the MY stage are similar to MDMs and circulating blood monocytes (respectively). Despite general similarities between the populations, fine comparative analyses revealed several phenotypic and transcriptomic differences between them (i.e., between iMCs and blood monocytes and between iMphs and MDMs) (van Wilgenburg et al., 2013; Alasoo et al., 2015; Zhang et al., 2015; Nenasheva et al., 2020). More importantly, the populations differ by their origin, HSC-independent and HSC-dependent, respectively. Thus, fundamental questions that arise are as follows: (i) Can iMCs be categorized as monocytes? (ii) Which mechanisms converge these populations that differentiate from different progenitors and in different microenvironment conditions?

### Variability of iMph Differentiation Protocols and the Identity of the Resulting Cells

Although the methods of iMph generation have been developed relatively recently, there is a great variability among them. The diversity concerns many culture conditions, of which the variety of exogenous factors used to drive the differentiation is probably the most striking, even in the same type of protocols. For example, the number of factors used in 2D-F protocols ranges from 3 (Yeung et al., 2017) to 11 (Cao et al., 2019; Konttinen et al., 2019), not to mention the variability in the exact list of factors, as well as the dose and timing of their application, which may all affect the cell differentiation process. The fact that despite this variability, all protocols resulted in successful iMph generation, raises the question whether some of the factors are surplus. Besides being different in their use of exogenous factors, the protocols also differ in many other culture conditions, including the method of mesoderm induction (spontaneous EB-based or factor-directed), the type of plastic, medium, the duration of iMC generation, and others. The question whether iMphs generated in these variable conditions are functionally and transcriptionally identical and how these conditions affect cell differentiation trajectories has not been addressed. Given the great potential of iMph application in the future, this will be important to know.

### Optimization of Existing Protocols

Because of the use of diverse culture conditions, iMph differentiation protocols differ by reproducibility, scalability, labor intensity, clinical applicability, and cost. Among the three main types of employed protocols, the EB-F_*HP*__+__*MY*_ type seems to have the best balance between the reproducibility (due to the factor-mediated control of the M/HE stage), clinical applicability (due to the use of xeno-free conditions and defined medium), efficacy (due to the continuous generation of iMphs), and cost (due to the use of only two cytokines, IL-3 and M-CSF, for HP/MY differentiation). EB-S protocols have the advantages of being relatively cheap and scalable and allowing a continuous iMph generation, but they are feeder- and serum-dependent and less reproducible. The prospects of 2D-F protocols (i.e., xeno-free conditions, defined medium, factor-dependent control of all differentiation stages, and reproducibility) are diminished by the use of multiple factors and one-off collection of iMphs, which decrease the cumulative cell yield and increase the protocol cost.

Considering the future prospects of iMph applications, an important task is to optimize the existing protocols so as to (i) increase iMph yield, (ii) observe the conditions necessary for clinical applications, and (iii) minimize iMph generation cost, i.e., to develop high-yield large-scale clinically applicable and economically suitable protocols. The first steps in this direction were focused on the scaling of the technique using bioreactor and other approaches (Ackermann et al., 2018; Gutbier et al., 2020). The other direction might be to determine the minimal list of factors sufficient to direct iMph differentiation. In this regard, the fact that iMCs may be generated using IL-3 and M-CSF only raises the question whether a similar (i.e., “HP+MY”) scheme may be applied to 2D-F protocols and, if so, whether it will allow continuous iMph generation in 2D cultures. Potentially, this could complement the benefits of 2D-F protocols with increased cell yields and a decreased cost. Overall, further progress requires an experimental comparison of existing protocols, the identification of minimal required conditions, and the development of standardized protocols for future iMph applications. The present review focusing on the variability of existing protocols constitutes only one step in this direction.

## Author Contributions

IL contributed to idea, analysis of the literature, manuscript writing and editing, and color table’s design. TG contributed to analysis of the literature, manuscript writing and editing, and table’s preparation. TN contributed to manuscript editing and table’s preparation. All authors contributed to the article and approved the submitted version.

## Conflict of Interest

The authors declare that the research was conducted in the absence of any commercial or financial relationships that could be construed as a potential conflict of interest.
